# Fragmented mitochondrial genomes of seal lice (family Echinophthiriidae) and gorilla louse (family Pthiridae): frequent minichromosomal splits and a host switch of lice between seals

**DOI:** 10.1186/s12864-022-08530-8

**Published:** 2022-04-08

**Authors:** Yalun Dong, Min Zhao, Renfu Shao

**Affiliations:** 1 Centre for Bioinnovation, University of the Sunshine Coast, 90 Sippy Downs Drive, Sippy Downs, Queensland 4556 Australia; 2 School of Science, Technology and Engineering, University of the Sunshine Coast, 90 Sippy Downs Drive, Sippy Downs, Queensland 4556 Australia

**Keywords:** Seal lice, Gorilla louse, Mitochondrial minichromosomes, Split and merge, Host switch

## Abstract

**Background:**

The mitochondrial (mt) genomes of 15 species of sucking lice from seven families have been studied to date. These louse species have highly dynamic, fragmented mt genomes that differ in the number of minichromosomes, the gene content, and gene order in a minichromosome between families and even between species of the same genus.

**Results:**

In the present study, we analyzed the publicly available data to understand mt genome fragmentation in seal lice (family Echinophthiriidae) and gorilla louse, *Pthirus gorillae* (family Pthiridae), in particular the role of minichromosome split and minichromosome merger in the evolution of fragmented mt genomes. We show that 1) at least three ancestral mt minichromosomes of sucking lice have split in the lineage leading to seal lice, 2) one minichromosome ancestral to primate lice has split in the lineage to the gorilla louse, and 3) two ancestral minichromosomes of seal lice have merged in the lineage to the northern fur seal louse. Minichromosome split occurred 15-16 times in total in the lineages leading to species in six families of sucking lice investigated. In contrast, minichromosome merger occurred only four times in the lineages leading to species in three families of sucking lice. Further, three ancestral mt minichromosomes of sucking lice have split multiple times independently in different lineages of sucking lice. Our analyses of mt karyotypes and gene sequences also indicate the possibility of a host switch of crabeater seal louse to Weddell seals.

**Conclusions:**

We conclude that: 1) minichromosome split contributes more than minichromosome merger in mt genome fragmentation of sucking lice, and 2) mt karyotype comparison helps understand the phylogenetic relationships between sucking louse species.

**Supplementary Information:**

The online version contains supplementary material available at 10.1186/s12864-022-08530-8.

## Background

Sucking lice (parvorder Anoplura) are obligate ectoparasites of eutherian mammals and feed exclusively on their host blood [1]. There are over 500 species of sucking lice, classified into 50 genera and 15 families [2]. Sucking lice have a flattened body to avoid host grooming, elongated mouthparts for blood-sucking, and sharp claws to climb and hold on host hair [3]. Unlike most animals, which have single-chromosome mitochondrial (mt) genomes [4], the sucking lice investigated to date have fragmented mt genomes, each with 9-20 minichromosomes [5–8]. Fifteen species of sucking lice from seven of the 15 families have been sequenced for complete or near complete mt genomes [5–13]. Human head louse (*Pediculus humanus capitis*, family Pediculidae) and human body louse (*Pediculus humanus corporis*) have the most fragmented mt genomes with 20 minichromosomes [5, 6], followed by chimpanzee louse (*Pediculus schaeffi*) with 18 minichromosomes [14]. Human pubic louse (*Pthirus pubis*, family Pthiridae) has 15 minichromosomes (*trnN* gene not identified) [6, 12]. Colobus louse (*Pedicinus badii*, family Pedicinidae) and macaque louse (*Pedicinus obtusus*) have 14 and 12 minichromosomes, respectively [12]. Guanaco louse (*Microthoradus praelongiceps*, family Microthoraciidae) has 12 minichromosomes [8]. Three rodent lice of the family Polyplacidae (*Polyplax asiatica*, *Polyplax spinulosa* and *Polyplax reclinata*) each have 11 minichromosomes [11, 13]. Two rodent lice of the family Hoplopleuridae (*Hoplopleura akanezumi* and *Hoplopleura kitti*) each have at least 11 minichromosomes with five and three genes not identified, respectively [10]. Wild pig louse (*Haematopinus apri*, family Haematopinidae), domestic pig louse (*Haematopinus suis*), and horse louse (*Haematopinus asini*) have the least fragmented mt genomes, each with nine minichromosomes [7, 9].

No species in the other eight families of sucking lice have been studied for their mt genome organization. These families are Echinophthiriidae (lice of seals), Enderleinellidae (lice of squirrels), Linognathidae (lice of cattle, sheep and goats), Hamophthiriidae (lice of colugos), Hybophthiridae (lice of aardvarks), Neolinognathidae (lice of elephant shrews), Pecaroecidae (lice of peccaries), and Ratemiidae (lice of horses, donkeys and zebras) [2, 15]. In the present study, we analyzed the publicly available Sequence Read Archive (SRA) data to understand the mt genome fragmentation in five species of seal lice (*Antarctophthirus carlinii*, *A. lobodontis*, *A. microchir*, *Lepidophthirus macrorhini, Proechinophthirus fluctus*) in the family Echinophthiriidae, and the gorilla louse (*Pthirus gorillae*) in the family Pthiridae. We also investigated the role of minichromosome split and merger in the evolution of the highly dynamic mt genome organization observed in sucking lice.

The family Echinophthiriidae has five genera and 13 species [2]. The genus *Antarctophthirus* has seven species, while the other four genera have one to two species. Seal lice are unique among parasitic lice in living in the marine environment [16, 17]. Ancestral seal lice were thought to be terrestrials but evolved unique morphology to adapt to marine life together with their hosts [15, 16, 18]. The family Pthiridae has only one genus *Pthirus* with two species: *Pthirus pubis* (human pubic louse) and *Pthirus gorillae* (gorilla louse) [2]. These two species diverged 3-4 million years ago (MYA), possibly due to host switch from gorillas to humans [19]. The mt genome of *Pthirus pubis* has been sequenced previously [6, 12]; however, the mt genome of *Pthirus gorillae* has not been studied. We find that: 1) seal lice differ substantially from sucking lice in other families in mt genome organization; 2) gorilla louse has a more fragmented mt genome than human pubic louse; 3) minichromosome split occurred in both lineages leading to seal lice and gorilla louse; 4) minichromosome merger occurred in the lineage to the northern fur seal louse; and 5) likely a host switch of louse occurred between crabeater seals and Weddell seals.

## Results

### Mitochondrial minichromosomes of *Lepidophthirus macrorhini* – louse of southern elephant seal (*Mirounga leonine*)

**Fig. 1:**
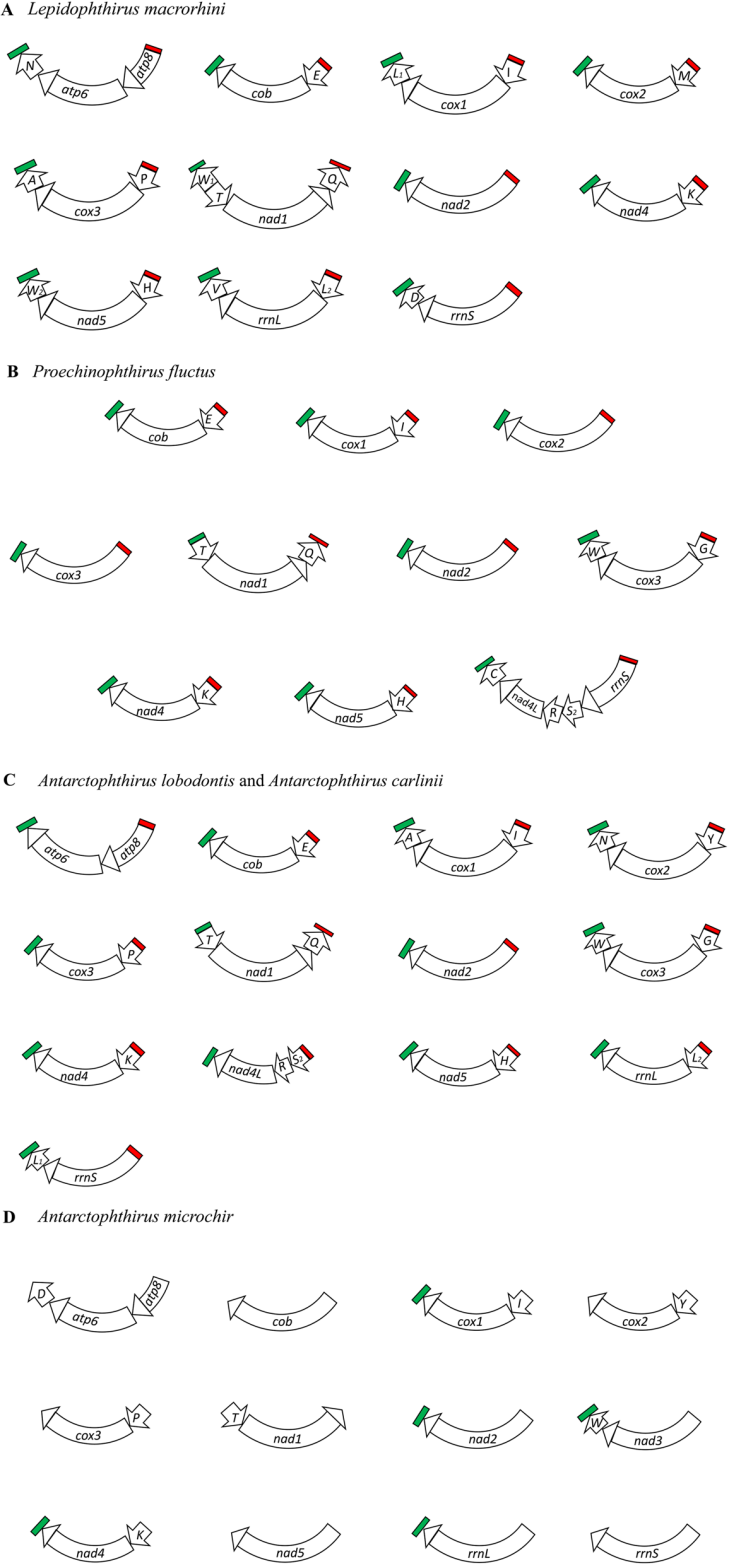
Mitochondrial genomes of five seal louse species: **A** southern elephant seal louse, *Lepidophthirus macrorhini*; **B** northern fur seal louse, *Proechinophthirus fluctus*; **C** Weddell seal louse, *Antarctophthirus carlinii,* and crabeater seal louse, *Antarctophthirus lobodontis*; and **D** Australian sea lion louse, *Antarctophthirus microchir*. Conserved AT-rich motifs are in red and conserved GC-rich motifs are in green. In *Antarctophthirus microchir*, AT-rich motifs were not identified and GC-rich motifs were identified in only five of the 12 minichromosomes. Names and transcription orientation of genes are indicated in the coding region. Gene names are: *atp6* and *atp8* for ATP synthase subunits 6 and 8; *cob* for cytochrome b; *cox1-3* for cytochrome c oxidase subunits 1-3, *nad1-5* and *nad4L* for NADH dehydrogenase subunits 1-5 and 4 L; *rrnS* and *rrnL* for small and large subunits of ribosomal RNA. tRNA genes are indicated with their single-letter abbreviations of the corresponding amino acids

The Illumina data of *Lepidophthirus macrorhini* (SRR5809351) from SRA database contains 47,978,079 paired-end sequence reads; each sequence read is 150 bp in size. We assembled these sequence reads and identified 27 of the 37 mt genes typical of bilateral animals. These genes are on 11 minichromosomes; each minichromosome contains a single protein-coding or rRNA gene with none or up to three tRNA genes except *atp8-atp6-N* minichromosome, which contains two protein-coding genes (Fig. 1A, Supplementary Table 1). The genes in all the minichromosomes have the same orientation of transcription relative to the non-coding regions except for *Q-nad1-T-W* minichromosome, in which three genes (underlined) have the opposite transcription orientation to all other genes (Fig. 1A, Supplementary Table 1). *trnW* gene were identified in two minichromosomes: *Q-nad1-T-W*_*1*_ minichromosome and *H-nad5-W*_*2*_ minichromosome. The pairwise identity between these two *trnW* genes is 50% (Supplementary Table 2); both have TCA anticodon and can form clove-leafed secondary structure (Supplementary Fig. 1). *trnW*_*1*_ has a higher identity to *trnW* of other seal lice (55.4 to 69.1%) than *trnW*_*2*_ has (42.6 to 55.1%), indicating *trnW*_*1*_ is more likely the original copy of *trnW*. However, it is unclear whether *trnW*_*2*_ is duplicated from *trnW*_*1*_ or from other tRNA genes (Supplementary Table 2). We obtained ~ 300 bp non-coding sequence both upstream and downstream from the coding region of each minichromosome (Supplementary Table 1). A conserved AT-rich (80.3%) motif (56 bp) and a conserved GC-rich (60.7%) motif (63 bp) were found in the non-coding sequences of all the minichromosomes (Fig. 1A, Supplementary Fig. 2). Ten mt genes were not identified in our analysis of *Lepidophthirus macrorhini*: *nad3, nad4L, nad6, trnC, trnF, trnG, trnR, trnS*_*1*_*, trnS*_*2*_ and *trnY.* The annotated mt minichromosomes of *Lepidophthirus macrorhini* were available in GenBank (accession numbers MW803094-104).

### Mitochondrial minichromosomes of *Proechinophthirus fluctus* – louse of northern fur seal (*Callorhinus ursinus*)

The Illumina data of *Proechinophthirus fluctus* (SRR5308138) from SRA database contains 5,819,192 paired-end sequence reads; each sequence read is 100 bp in length. We assembled these sequence reads and identified 22 of the 37 typical mt genes. These genes are on 10 minichromosomes; each minichromosome contains a single protein-coding or rRNA gene with none or up to three tRNA genes (Fig. 1B, Supplementary Table 3). The genes in all the minichromosomes have the same orientation of transcription relative to the non-coding regions except for *Q-nad1-T* minichromosome; these three genes have the opposite transcription orientation to all other genes (Fig. 1B). We obtained ~ 200 bp non-coding sequence both upstream and downstream from the coding region of each minichromosome (Supplementary Table 3). A conserved AT-rich (71.8%) motif (85 bp) and a conserved GC-rich (61.7%) motif (81 bp) were found in the non-coding sequences of all the minichromosomes (Fig. 1B, Supplementary Fig. 3). Fifteen mt genes were not identified in our analysis of the SRA data of *Proechinophthirus fluctus*: *atp6, atp8, nad6, rrnL, trnA, trnD, trnF, trnL*_*1*_, *trnL*_*2*_, *trnM, trnN, trnP, trnS*_*1*_, *trnV* and *trnY*. The annotated mt minichromosomes of *Proechinophthirus fluctus* were available in GenBank (accession numbers MW803105-114).

### Mitochondrial minichromosomes of *Antarctophthirus carlinii* - louse of Weddell seal (*Leptonychotes weddelli*), and *Antarctophthirus lobodontis* - louse of crabeater seal (*Lobodon carcinophagus*)

The Illumina data of *Antarctophthirus carlinii* (SRR5809348) and *Antarctophthirus lobodontis* (SRR5809349) from SRA database contains 39,054,456 and 45,005,741 paired-end sequence reads respectively; each sequence read is 150 bp. We assembled these sequence reads and identified 30 of the 37 typical mt genes on nine minichromosomes in each species (Fig. 1C). Each minichromosome contains a single protein-coding gene with none or up to two tRNA genes (Fig. 1C, Supplementary Tables 4 and 5). The gene content and gene arrangement in each mt minichromosome are the same between these two species; furthermore, the identity of each homologous gene is 94.3 to 100% between the two species. The genes in all the minichromosomes have the same orientation of transcription relative to the non-coding regions except for *Q-nad1-T* minichromosome, in which the three genes have the opposite transcription orientation to all other genes (Fig. 1C). We obtained ~ 300 bp non-coding sequence both upstream and downstream from the coding region of each minichromosome (Supplementary Tables 4 and 5). In *Antarctophthirus carlinii,* a conserved AT-rich (60%) motif (60 bp) and a conserved GC-rich (67.4%) motif (46 bp) were found in the non-coding sequences of all the minichromosomes (Supplementary Fig. 4). Conserved AT-rich (68.7%) motif (65 bp) and GC-rich (64.9%) motif (37 bp) were also found in *Antarctophthirus lobodontis* (Supplementary Fig. 5). Seven mt genes were not identified in our analysis of the SRA data of these two *Antarctophthirus* species: *nad6, trnC, trnD, trnF, trnM, trnS*_*1*_ and *trnV*. The annotated mt minichromosomes of *Antarctophthirus carlinii* (accession numbers MW803073-81) and *Antarctophthirus lobodontis* (accession numbers MW803064-72) were available in GenBank.

### Mitochondrial minichromosomes of *Antarctophthirus microchir* – louse of Australian sea lion (*Neophoca cinerea*)

The Illumina data of *Antarctophthirus microchir* (SRR5809347) from SRA database contains 43,650,933 paired-end sequence reads. Each sequence read is 150 bp in size. We assembled these sequence reads and identified 20 of the 37 typical mt genes. These genes are on 12 minichromosomes; each minichromosome contains a single protein-coding or rRNA gene with none or one tRNA gene except *atp8-atp6-D* minichromosome (Fig. 1D, Supplementary Table 6). We obtained 250 bp non-coding sequence both upstream and downstream from the coding region of each minichromosome (Supplementary Table 6). We identified a conserved GC-rich motif in the non-coding sequences of five of the 12 minichromosomes; however, we were unable to identify conserved AT-rich motif in the non-coding sequences of any minichromosomes (Fig. 1D, Supplementary Table 6, Supplementary Fig. 6). Full-length non-coding region sequences are needed to investigate whether the unidentified conserved motifs are in the middle section of the non-coding regions of *Antarctophthirus microchir*. Seventeen mt genes were not identified in our analysis of the SRA data of *Antarctophthirus microchir*: *nad4L, nad6, trnA, trnC, trnE, trnF, trnG, trnH, trnL*_*1*_, *trnL*_*2*_, *trnM, trnN, trnQ, trnR, trnS*_*1*_, *trnS*_*2*_ and *trnV*. The annotated mt minichromosomes of *Antarctophthirus microchir* were available in GenBank (accession numbers MW803082-93).

### Mitochondrial minichromosomes of *Pthirus gorillae* – louse of Western gorilla (*Gorilla gorilla*)

**Fig. 2:**
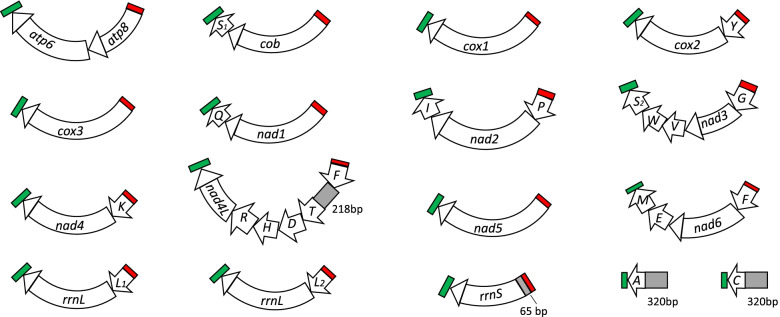
Mitochondrial genome of the gorilla louse, *Pthirus gorillae.* Conserved AT-rich motifs are in red; conserved GC-rich motifs are in green; and degenerate genes are in grey. Names and transcription orientation of genes are indicated in the coding region. *nad6* is the gene for NADH dehydrogenase subunit 6. Names of other genes are described in Fig. 1 legend

**Fig. 3:**
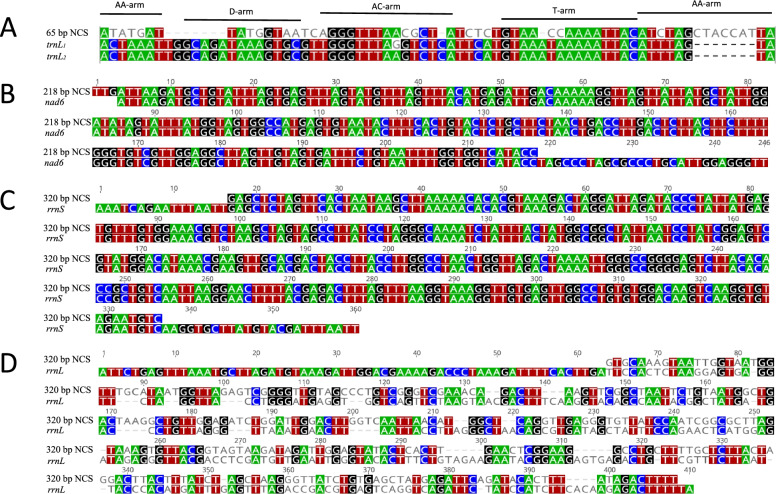
Sequence alignment of degenerate genes with their corresponding full-length genes. **A**: the 65-bp non-coding sequence (NCS) in *rrnS* minichromosome has 58.5% identity to *trnL1* gene and 60% identity to *trnL2* gene. **B**: The 218-bp intergenic NCS in *F-T-D-H-R-nad4L* minichromosome has 215 bp identical to a 5′ section of *nad6* gene (nucleotide 1 to 215). **C** The 320-bp NCS in *A* minichromosome is identical to a middle section of *rrnS* gene (nucleotide 246 to 565). **D** The 320-bp NCS in *C* minichromosome has 53.3% identity to 3′ section of *rrnL* gene (nucleotide 838 to 1189)

The Illumina data of *Pthirus gorillae* (SRR5088474) from SRA database contains 60,425,294 paired-end sequence reads; each sequence read is 160 bp in length. We assembled these sequence reads and identified 36 of the 37 typical mt genes; these genes are on 17 minichromosomes (Fig. 2). *trnN* was the only gene not identified in our analysis. Fourteen of the 17 minichromosomes contain a single protein-coding or rRNA gene with none or up to five tRNA genes. Of the other three minichromosomes, *atp8-atp6* minichromosome contains no tRNA gene whereas *trnA* minichromosome and *trnC* minichromosome each have only a tRNA gene (Fig. 2, Supplementary Table 7). The genes in all the minichromosomes have the same orientation of transcription relative to the non-coding regions (Fig. 2). A 65-bp non-coding sequence upstream from *rrnS* has 58.5% identity to *trnL*_*1*_ and 60% identity to *trnL*_*2*_ (Fig. 3A). As *trnL* gene is upstream from *rrnS* in the human pubic louse (*Pthirus pubis*), human head louse (*Pediculus humanus capitis*) and human body louse (*Pediculus humanus corporis*) [5], the 65 bp non-coding sequence upstream from *rrnS* in *Pthirus gorillae* is very likely a degenerate *trnL* gene. Three other regions are likely degenerate genes too: 1) a 218-bp sequence between *trnF* and *trnT* has 100% identity with a 5′ section of *nad6* (Fig. 3B); 2) a 320-bp sequence upstream from *trnA* has 100% identity with a middle section of *rrnS* (Fig. 3C); and 3) a 320-bp sequence upstream from *trnC* has 53.3% identity to 3′ section of *rrnL* (Fig. 3D). We obtained ~ 320 bp non-coding sequence both upstream and downstream from the coding region of each minichromosome (Supplementary Table 7). A conserved AT-rich (76.6%) motif (96 bp) was found in the non-coding sequences of all the minichromosomes except *trnA* minichromosome and *trnC* minichromosome (Fig. 2; Supplementary Fig. 7). A GC-rich (76%) motif (25 bp) was found in the non-coding sequences of all the minichromosomes (Fig. 2; Supplementary Fig. 7). The annotated mt minichromosomes of *Pthirus gorillae* are available in GenBank (accession numbers MW803115-131).

### Phylogeny of sucking lice based on mitochondrial gene sequences

**Fig. 4:**
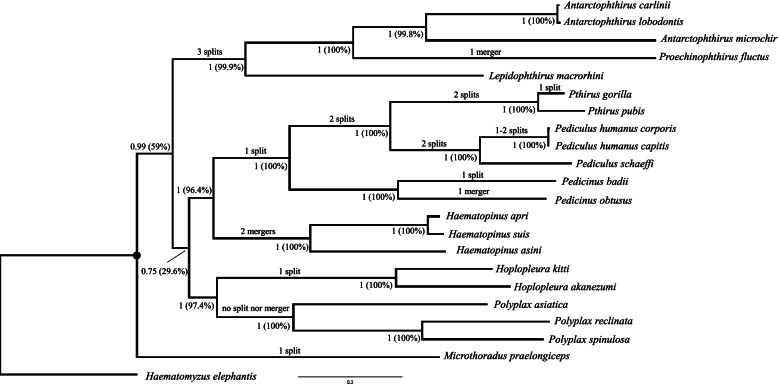
Bayesian phylogenetic tree inferred from the nucleotide sequences of five mitochondrial protein-coding genes (*cob*, *cox1*, *cox2*, *cox3*, *nad4*) of 21 species of sucking lice (Anoplura). The elephant louse, *Haematomyzus elephantis*, was used as the outgroup. Bayesian posterior probability (Bpp) values were indicated near nodes followed by bootstrap support values (in brackets) from maximum likelihood (ML) tree (Supplementary Fig. 8)

We reconstructed the phylogeny of sucking lice (Anoplura) to assist the inference of ancestral mt karyotype of seal lice (family Echinophthiriidae). Both the Bayesian and maximum likelihood (ML) trees strongly support the monophyly of Echinophthiriidae and each of the other seven families of sucking lice (Fig. 4; Supplementary Fig. 8). The relationships among the five species of seal lice are resolved with strong support. The three *Antarctophthirus* species are most closely related to each other, among which the Weddell seal louse (*Antarctophthirus carlinii*) and the crabeater seal louse (*Antarctophthirus lobodontis*) are sister to one another, both having very short branch length relative to that of the Australian sea lion louse (*Antarctophthirus microchir*). The *Antarctophthirus* species are more closely related to the northern fur seal louse (*Proechinophthirus fluctus*) than to the southern elephant seal louse (*Lepidophthirus macrorhini*) (Fig. 4; Supplementary Fig. 8).

The monophyly of primate lice (families Pediculidae, Pthiridae and Pedicinidae) is strongly supported and the relationships among the primate lice are resolved with strong support (Fig. 4; Supplementary Fig. 8). The gorilla louse (*Pthirus gorillae*) is most closely related to the human pubic louse (*Pthirus pubis*); the *Pthirus* species are more closely related to the *Pediculus* species of human and chimpanzee than to the *Pedicinus* species of monkeys. There is support in both Bayesian and ML trees for: 1) the primate lice to be most closely related to the pig and horse lice (family Haematopinidae); and 2) the rodent lice in the families Polyplacidae and Hoplopleuridae to be closely related. There is support in the Bayesian tree but not in the ML tree for: 1) Pthiridae, Pediculidae, Pedicinidae, Haematopinidae, Hoplopleuridae and Polyplacidae to be more closely related to each other than to Microthoraciidae or Echinophthiriidae; and 2) Echinophthiriidae to be more closely related the group that contains Pthiridae, Pediculidae, Pedicinidae, Haematopinidae, Hoplopleuridae and Polyplacidae than to Microthoraciidae (Fig. 4; Supplementary Fig. 8).

### Inferred ancestral mitochondrial karyotype of the seal lice

**Fig. 5:**
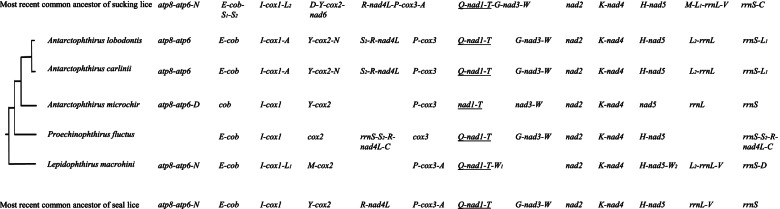
Inference of the ancestral mitochondrial karyotype of seal lice. Minichromosomal characters were inferred to be ancestral to seal lice if a character was present in: 1) one or more of the five species of seal lice and also in the MRCA of sucking lice; or 2) all the five species of seal lice. Gene names were described in Fig. 1 legend. Genes underlined have opposite transcription orientation to those not underlined

The mt minichromosomal information and the sucking louse phylogeny reported above allowed us to infer the partial mt karyotype of the most recent common ancestor of the five species of seal lice. There were at least 13 minichromosomes in the ancestral mt karyotype of these seal lice (Fig. 5). The position and arrangement of 12 of the 13 mt protein-coding genes, two rRNA genes and 14 of the 22 tRNA genes in the ancestral mt karyotype can be inferred based on the minichromosomal information available from the five species of seal lice. The position and arrangement of *nad6* and eight tRNA genes, however, cannot be inferred due to: 1) *nad6* was not identified in any of the five species of seal lice; and 2) the eight tRNA genes are very variable in their arrangement among the five species of seal lice (Fig. 5).

## Discussion

### Mitochondrial minichromosome split occurred more frequently than minichromosome merger in the lineages leading to seal lice and gorilla louse

Previous studies indicated that both split and merger of mt minichromosomes occurred in sucking lice and were responsible to a large degree to their highly dynamic mt genome organization [8, 12, 13]. Prior to the current study, split of mt minichromosomes was observed in species from five of the seven families of sucking lice studied: Pediculidae, Pthiridae, Pedicinidae, Microthoraciidae, and Hoplopleuridae, whereas merger of mt minichromosomes was observed in species from two families: Haematopinidae and Pedicinidae [7–9, 12]. Neither split nor merger of mt minichromosome occurred in the family Polyplacidae [8, 11, 13]. The mt genome organization of other eight families of sucking lice was unknown prior to the current study: Echinophthiriidae (lice of seals), Enderleinellidae (lice of squirrels), Linognathidae (lice of cattle, sheep, and goats), Hamophthiriidae (lice of colugos), Hybophthiridae (lice of aardvarks), Neolinognathidae (lice of elephant shrews), Pecaroecidae (lice of peccaries) and Ratemiidae (lice of horses, donkeys, and zebras).

We showed in the current study that at least three ancestral mt minichromosomes of the sucking lice [8] have split further in the lineage leading to seal lice (Echinophthiriidae): 1) *D-Y-cox2-nad6* minichromosome, 2) *R-nad4L-P-cox3-A* minichromosome, and 3) *Q-nad1-T-G-nad3-W* minichromosome (Fig. 5). In all the five species of seal lice, *cox2* has its own minichromosome not shared with any other protein-coding gene(s), indicating the split of *D-Y-cox2-nad6* minichromosome occurred in the most recent common ancestor (MRCA) of these seal lice although we could not identify *nad6* in our SRA data analyses (Fig. 5). Similarly, *R-nad4L-P-cox3-A* minichromosome and *Q-nad1-T-G-nad3-W* minichromosome also split in the MRCA of seal lice. *nad4L* has its minichromosome not shared with any other protein-coding gene(s) in *Antarctophthirus lobodontis* and *Antarctophthirus carlinii*, and *cox3* has its minichromosome not shared with any other protein-coding gene(s) in all the five species of seal lice (Fig. 5). *nad1* has its minichromosome not shared with any other protein-coding gene(s) in all the five species of seal lice; furthermore, *nad3* has its minichromosomes not shared with any other protein-coding gene(s) in *Antarctophthirus microchir* and *Proechinophthirus fluctus* (Fig. 5). We also observed a merger event in the lineage to *Proechinophthirus fluctus* between two ancestral mt minichromosomes inferred for the seal lice: *R-nad4L* and *rrnS* (Fig. 5).

The family Pthiridae has a single genus and two species: gorilla louse (*Pthirus gorillae)* and human pubic louse (*Pthirus pubis*); these two species diverged 3-4 MYA [19]. Previous studies showed that the mt genome of *Pthirus pubis* has 15 minichromosomes with *trnN* gene still not identified [6, 12]. In the current study, we showed that the mt genome of *Pthirus gorilla* has 17 minichromosomes (*trnN* not identified either) and is more fragmented than that of *Pthirus pubis* (Fig. 2). *cox3-A* minichromosome is ancestral to primate lice and is retained in *Pthirus pubis* [6]; however, this minichromosome is split into two in *Pthirus gorilla*: one minichromosome contains *cox3* and other has *trnA* (Fig. 2).

### The frequency of split and merger varies among different families of sucking lice, so does the type of minichromosomes that split and merge

Shao et al. [8] proposed that split and merger of mt minichromosomes contributed to the complex and dynamic mt genome organization observed in sucking lice. Shao et al. [8] reported that: 1) split of mt minichromosomes occurred in sucking louse species from four families after their divergence from the MRCA of sucking lice: Hoplopleuridae, Pthiridae, Pediculidae and Microthoraciidae; 2) merger of mt minichromosomes occurred in species from the family Haematopinidae; and 3) no split nor merger occurred in species from the family Polyplacidae, which was also confirmed in Dong et al. [13]. Fu et al. [12] reported that both split and merger of mt minichromosomes occurred in the family Pedicinidae: split of a minichromosome occurred in the colobus louse *Pedicinus badii* whereas merger of minichromosomes occurred in the macaque louse *Pedicinus obtusus*, relative to the MRCA of higher primate lice. The other eight families of sucking lice were unknown previously for their mt genome organization: Echinophthiriidae (lice of seals), Enderleinellidae (lice of squirrels), Linognathidae (lice of cattle, sheep and goats), Hamophthiriidae (lice of colugos), Hybophthiridae (lice of aardvarks), Neolinognathidae (lice of elephant shrews), Pecaroecidae (lice of peccaries) and Ratemiidae (lice of horses, donkeys and zebras) [2, 15]. In the present study, we showed that split of three minichromosomes occurred in seal lice of the family Echinophthiriidae relative to the MRCA of sucking lice, and merger of two minichromosomes in the lineage leading to the northern fur seal louse, *Proechinophthirus fluctus* (Fig. 4). We also showed that split of a minichromosome occurred in the gorilla louse, *Pthirus gorilla*, after its divergence from the human pubic louse, *Pthirus pubis*.

It is apparent that split of minichromosomes occurs much more frequently and in more families than merger of minichromosomes in sucking lice (Fig. 4). Furthermore, the frequency of minichromosome split varies from family to family with the two families of great ape lice (Pthiridae, Pediculidae) having the highest number of minichromosome split (8-9 split events) but no split in Haematopinidae and Polyplacidae (Fig. 4). It is noteworthy that a few ancestral minichromosomes to sucking lice split multiple times independently in lineages leading to different families. Shao et al. [8] showed that: 1) *D-Y-cox2-nad6* minichromosome split twice independently, once in Hoplopleuridae, and another time in Pediculidae and Pthiridae; and 2) *Q-nad1-T-G-nad3-W* minichromosome split twice independently, once in Microthoraciidae, and another time in Pediculidae and Pthiridae. In the current study, we found that these two minichromosomes also split independently in seal lice (Echinophthiriidae). Furthermore, *R-nad4L-P-cox3-A* minichromosome, which split in Pediculidae and Pthiridae [8], also split independently in seal lice (Echinophthiriidae).

Much rarer than minichromosome split, minichromosome merger was seen only in species from three families: pig lice and horse louse (Haematopinidae) [8], macaque louse (Pedicinidae) [12], and northern fur seal louse (Echinophthiriidae). In the pig lice and horse louse, two ancestral minichromosomes to sucking lice, *atp8-atp6-trnN* and *trnK-nad4* merged; two other ancestral minichromosomes, *trnI-cox1-trnL*_*2*_ and *nad2* also merged [8]. In the macaque louse, a single merge event occurred between two ancestral minichromosomes to higher primate lice that contain *cox2* and *nad2* genes respectively. In the northern fur seal louse, a single merger event occurred between two minichromosomes ancestral to seal lice that contain *nad4L* and *rrnS* genes respectively (Fig. 5). It is noteworthy that *nad2* minichromosome was involved in two separate merger events whereas the other five minichromosomes were each involved only in a single merger event.

### The very high mitochondrial gene identity shared between *Antarctophthirus carlinii* and *Antarctophthirus lobodontis* does not support them as separate species

The crabeater seal louse, *Antarctophthirus lobodontis*, was described by Enderlein [20] and redescribed by Leonardi et al. [21]; the Weddell seal louse, *Antarctophthirus carlinii*, was described by Leonardi et al. [22]. These two species are very similar in morphology [21, 22]. Leonardi et al. [21] provided two morphological characters to differentiate between these two species: 1) *Antarctophthirus carlinii* has six dorsal posterior long hairs (four marginal and two principal) around the posterior border of the head while *Antarctophthirus lobodontis* has only four marginal long hairs around the posterior border of the head; and 2) *Antarctophthirus lobodontis* has a line of eight spines in the basis of the head and three hairs above the last row of four spines. As reported above, *Antarctophthirus carlinii* and *Antarctophthirus lobodontis* have identical mt karyotypes (Fig. 1C), indicating a very close relationship between them. Furthermore, *Antarctophthirus carlinii* and *Antarctophthirus lobodontis* share very high identity of mt genes ranging from 94.3 to 100% (average 99.03%) (Supplementary Table 8). We also obtained and compared the partial sequences of three nuclear genes between *Antarctophthirus carlinii* and *Antarctophthirus lobodontis*. The sequences of their *elongation factor 1-α* gene (182 bp) are 100% identical; their *18S* sequences (1054 bp) and *28S* rRNA gene sequences (2295 bp) have 99.3 and 99.1% identities respectively (Supplementary Fig. 9). Previous studies showed that mt genes of parasitic lice evolved much faster than their hosts. Thus, the identities of mt genes are much lower between parasitic lice than between their hosts [23–26]. This is, however, not the case for *Antarctophthirus carlinii* and *Antarctophthirus lobodontis*. The mt gene identities between these lice are 4.11% higher on average than that between their hosts, the Weddell seal (*Leptonychotes weddellii*) and the crabeater seal (*Lobodon carcinophagus*) - 90.8 to 100% identities with an average of 94.99% (Supplementary Table 9). The low genetic divergence between *Antarctophthirus carlinii* and *Antarctophthirus lobodontis* does not support them as separate species; more likely they are two subspecies instead of two species. Leonardi et al. [18] also discussed the possibility that *Antarctophthirus carlinii* and *Antarctophthirus lobodontis* were the same species based on the low genetic divergence between them. However, based on cophylogenetic analysis, Leonardi et al. [18] concluded that *Antarctophthirus carlinii* and *Antarctophthirus lobodontis* co-speciated with their hosts thus were different species; this conclusion was not supported by the lower genetic divergence between *Antarctophthirus carlinii* and *Antarctophthirus lobodontis* than between their hosts. A relatively recent host switch between the Weddell seal and the crabeater seal is a more plausible explanation for the low genetic divergence observed between *Antarctophthirus carlinii* and *Antarctophthirus lobodontis* than co-speciation of *Antarctophthirus carlinii* and *Antarctophthirus lobodontis* with their hosts [18]. Both Weddell seals and crabeater seals have circumpolar distributions in the Antarctic and share the same microhabitats such as land-fast ice and pack ice when they rest and breed [27, 28]. This could provide ample opportunities for the parasitic lice of one seal species to explore and switch to another seal species. It is unclear to us, however, which seal is the original host and which seal is the new host. One possibility is that the host switch occurred from crabeater seal to Weddell seal based on the observation that crabeater seal hosts only one louse species (*Antarctophthirus lobodontis*) whereas Weddell seal hosts two louse species (*Antarctophthirus carlinii* and *Antarctophthirus ogmorhini*) [2]. Both crabeater seal and Weddell seal are in the tribe Lobodontini (subfamily Monachinae), together with Ross seal (*Ommatophoca rossi*i) and leopard seal (*Hydrurga leptonyx*) [2]. Weddell seal is the only species in the tribe Lobodontini that hosts two louse species; the other three seal species each host one louse species only [2]. Further studies on the phylogeny of all louse species of Lobodontini seals should reveal more on the host-parasite relationships in this tribe.

## Conclusion

In this study, we assembled the mt genomes of five species of seal lice and the gorilla louse, and conducted phylogenetic analysis of sucking lice from eight families. We inferred the ancestral mt karyotype of seal lice and analyzed the frequency of mt minichromosomal split and merger among sucking lice. Mt karyotype comparison, gene sequence analysis and phylogenetic analysis all indicated that the crabeater seal louse, *Antarctophthirus lobodontis*, and the Weddell seal louse, *Antarctophthirus carlinii*, are likely two subspecies instead of two species, and the possibility of a host switch of crabeater seal louse to Weddell seals. We showed that at least three ancestral mt minichromosomes of sucking lice have split in the lineage leading to seal lice, one minichromosome ancestral to primate lice has split in the lineage leading to gorilla louse, and two ancestral minichromosomes of seal lice have merged in the lineage to the northern fur seal louse. Split of mt minichromosomes occurred 15-16 times in total in the lineages leading to six families of sucking lice studied so far whereas merger of minichromosomes occurred only four times in the lineages leading to three families of sucking lice. Furthermore, three ancestral minichromosomes to sucking lice, *D-Y-cox2-nad6*, *Q-nad1-T-G-nad3-W* and *R-nad4L-P-cox3-A*, have split independently in different lineages of sucking lice. We conclude that: 1) minichromosome split contributes much more than minichromosome merger in mt genome fragmentation of sucking lice, and 2) mt karyotype comparison, in conjunction with gene sequence analysis, helps understand the relationship between sucking louse species.

## Materials and methods

### Retrieval and assembly of Sequence Read Archive (SRA) data of seal lice and gorilla louse

The SRA data of five species of seal lice (*Antarctophthirus carlinii*; *Antarctophthirus lobodontis*, *Antarctophthirus microchir*, *Lepidophthirus macrorhini* and *Proechinophthirus fluctus*) and the gorilla louse (*Pthirus gorillae*) were retrieved from NCBI SRA database (https://www.ncbi.nlm.nih.gov/sra/). These SRA data were produced with Illumina platforms in whole genome sequencing projects and were deposited by researchers in Kevin Johnson’s group at the University of Illinois, Urbana-Champaign [18]. The whole dataset of sequence-reads of each species was imported into and assembled in Geneious 11.0.2 [29]. A subset of 15,000 sequence reads in each dataset was extracted and assembled de novo to obtain seed contigs for mt gene search; the number of contigs to be displayed was set at 100. The consensus sequences of these 100 contigs were searched in a batch in NCBI “Non-redundant protein sequences (NR)” database using BLASTx and default parameters to identify protein gene sequence matches [30]. The consensus sequences that matched significantly (E-value < 10^− 15^) to the mt protein sequences of sucking lice (parvorder Anoplura) in the NR database were used as reference sequences to assemble the full-length coding region and its adjacent non-coding regions (200-320 bp upstream and downstream) of each mt minichromosome; the entire set of SRA sequence-reads of each species was explored in coding and non-coding region assembly. We did not attempt to assemble the full-length non-coding region of each minichromosome with SRA data because: 1) the non-coding region is highly similar in sequence among different mt minichromosomes of a sucking louse species [5–13], and 2) the SRA Illumina sequence reads are too short (100-160 bp each) for such attempt. The key assembly parameters were: 1) minimum overlap 60 bp for *Proechinophthirus fluctus* (SRA sequence reads 100 bp each) and 100 bp for other four species of seal lice (SRA sequence reads 150 bp each) and gorilla louse (SRA sequence reads 160 bp each); and 2) minimum identity 95%. Because of the high sequence similarity among the non-coding regions of different minichromosomes within a species, once two or more minichromosomes were assembled, the conserved sequence motifs including the hallmark AT-rich motif and GC-rich motif in the non-coding regions were identified by sequence alignment and used as references to identify and assemble the remaining minichromosomes. An AT-rich motif upstream from coding region and a GC-rich motif downstream from coding region are present in the mt minichromosomes of all sucking lice and the chewing lice of eutherian mammals sequenced to date [5–14, 31, 32]. The steps described above were repeated multiple times for each species until no more additional mt genes could be found. The protein-coding genes and rRNA genes in mt minichromosomes were identified by BLAST search in NCBI database; tRNA genes were identified by tRNA-scan [33] and ARWEN [34].

For *Antarctophthirus carlinii* and *Antarctophthirus lobodontis*, their partial *elongation factor 1-α* gene sequence, *18S* and *28S* rRNA gene sequences were generated by Illumina sequence read assembly, using the available sequences of *Haematopinus eurysterunus* (accession numbers HM171457 and HM171381) and *Echinophthirus horridus* (accession number KX810111) as initial reference sequences. We used Geneious 11.0.2 [29] and started with *medium sensitivity* in reference assembly to find the most conserved regions of these three genes. Then we used the SRA sequence reads that were mapped to the conserved regions as references to initiate and extend the contigs for *elongation factor 1-α*, *18S* rRNA and *28S* rRNA genes of *Antarctophthirus carlinii* and *Antarctophthirus lobodontis.* These genes were then verified by BLAST search in NCBI database.

### Phylogenetic analyses

Twenty-two species of parasitic lice (Supplementary Table 10) were included in our phylogenetic analysis: 1) the five species of seal lice and the gorilla louse reported in the present study; 2) 15 sucking louse species reported in previous studies; and 3) the elephant louse (*Haematomyzus elephantis*) as the outgroup. The sequences of five mt protein-coding genes (*cob*, *cox1*, *cox2*, *cox3*, *nad4*) were used in our phylogenetic analysis. These five genes were aligned individually using MAFFT 7.471, then concatenated into a single file after removing the poorly aligned sites using Gblocks 0.91b. Two methods were used in our phylogenetic analysis: 1) maximum likelihood (ML) with IQ-Tree [35], and 2) Bayesian inference method (BI) with MrBayes 3.2.6 [36]. Model test were done in IQ-TREE [37] and the best-fit model is TIM + F + R4. For ML analysis, the bootstrap replicates were set at 1000. For BI analyses, four independent Markov Chains were run for 5 million MCMC generations, sampling a tree every 100 generations. This analysis was run until the average standard deviation of split frequencies was lower than 0.001. The ML tree and BI tree were drawn with Figtree v1.4.3 (http://tree.bio.ed.ac.uk/software/figtree).

### Inferring the ancestral mitochondrial karyotype of seal lice

To infer the ancestral mt karyotype of seal lice, we used a parsimony method described in Shao et al. [8]. The ancestral mt karyotype of seal lice was inferred based on a comparison of mt minichromosomal characters between the five species of seal lice and the inferred most recent common ancestor (MRCA) of sucking lice. We inferred a minichromosomal character to be ancestral to seal lice if: 1) the character was present in one or more of the five species of seal lice and also in the MRCA of sucking lice; or 2) the character is present in all the five species of seal lice. For example, the character *E-cob* in a minichromosome is present in four of the five seal louse species and also in the MRCA of sucking lice (Fig. 5). Thus, *E-cob* in a minichromosome is inferred to be ancestral to seal lice. The MRCA of sucking lice also has *S*_*1*_-*S*_*2*_ in the same minichromosome with *E-cob*; these two tRNA genes are most likely translocated to other minichromosome(s) in the MRCA of seal lice as they are not with *E-cob* in any of the five seal louse species (Fig. 5). Taking another example, *K-nad4* in a minichromosome is present in all the five seal louse species thus it is inferred to be ancestral to seal lice (Fig. 5). In this case, *K-nad4* is also present in the MRCA of sucking lice, which reinforces the inference of *K-nad4* minichromosome to be ancestral to seal lice.

## Supplementary Information

**Additional file 1: Supplementary Fig. 1.** Secondary structure inferred with tRNA-Scan [33] from *trnW1* and *trnW2* gene sequences of the southern elephant seal louse, *Lepidophthirus macrorhini*. **Supplementary Fig. 2.** Conserved non-coding AT-rich motifs and GC-rich motifs among the mitochondrial minichromosomes of the southern elephant seal louse, *Lepidophthirus macrorhini.* **Supplementary Fig. 3.** Conserved non-coding AT-rich motifs and GC-rich motifs among the mitochondrial minichromosomes of the northern fur seal louse, *Proechinophthirus fluctus*. **Supplementary Fig. 4.** Conserved non-coding AT-rich motifs and GC-rich motifs among the mitochondrial minichromosomes of the Weddell seal louse, *Antarctophthirus carlinii.* **Supplementary Fig. 5.** Conserved non-coding AT-rich motifs and GC-rich motifs among the mitochondrial minichromosomes of the crabeater seal louse, *Antarctophthirus lobodontis.* **Supplementary Fig. 6.** Conserved non-coding GC-rich motifs among the mitochondrial minichromosomes of the Australian sea lion louse, *Antarctophthirus microchir*. **Supplementary Fig. 7.** Conserved non-coding AT-rich motifs and GC-rich motifs among the mitochondrial minichromosomes of the gorilla louse, *Pthirus gorillae.* **Supplementary Fig. 8.** Phylogenetic relationships among 21 species of sucking lice (Anoplura) inferred by maximum likelihood (ML) analysis of nucleotide sequences of five mitochondrial protein-coding genes. The elephant louse, *Haematomyzus elephantis*, was used as the outgroup. The ultrafast bootstrap support (%) / SH-aLRT support (%) were indicated near each node. **Supplementary Fig. 9.** Alignment of partial *18S* rRNA gene, *28S* rRNA gene and *ef1-α* gene sequences between *Antarctophthirus carlinii* and *Antarctophthirus lobodontis*. **Supplementary Table 1.** Mitochondrial minichromosomes of *Lepidophthirus macrorhini -* louse of southern elephant seal (*Mirounga leonine*). **Supplementary Table 2.** Sequence identities between *trnW1* and *trnW2* of the southern elephant seal louse, *Lepidophthirus macrorhini*, between *trnW* genes of *Lepidophthirus macrorhini* and other seal lice, and between *trnW* genes and other tRNA genes of *Lepidophthirus macrorhini*. Identities were generated with ClustalW in Geneious [29]: cost matrix IUB, gap open cost 15, gap extend cost 6.66. **Supplementary Table 3.** Mitochondrial minichromosomes of *Proechinophthirus fluctus -* louse of northern fur seal (*Callorhinus ursinus*). **Supplementary Table 4.** Mitochondrial minichromosomes of *Antarctophthirus carlinii* - louse of Weddell seal (*Leptonychotes weddelli*). **Supplementary Table 5.** Mitochondrial minichromosomes of *Antarctophthirus lobodontis* - louse of crabeater seal (*Lobodon carcinophagus*). **Supplementary Table 6.** Mitochondrial minichromosomes of *Antarctophthirus microchir* – louse of Australian sea lion (*Neophoca cinerea*). **Supplementary Table 7.** Mitochondrial minichromosomes of *Pthirus gorilla* – louse of western gorilla (*Gorilla gorilla*). **Supplementary Table 8.** Sequence identities between *Antarctophthirus carlinii* (louse of Weddell seal, *Leptonychotes weddelli*) and *Antarctophthirus lobodontis* (louse of crabeater seal, *Lobodon carcinophagus*). **Supplementary Table 9.** Sequence identities between Weddell seal (*Leptonychotes weddelli*) and crabeater seal (*Lobodon carcinophagus*). **Supplementary Table 10.** Species of parasitic lice included in the phylogenetic analyses in this study.

## Data Availability

Sequence data generated in this study are available in NCBI **(**accession numbers MW803064-803131).

## Abbreviations

mt: Mitodhindrial
*atp6* and *atp8*: Genes for ATP synthase subunits 6 and 8
bp: Base pair
*cob*: Gene for cytochrome b
*cox1*, *cox2* and *cox3*: Genes for cytochrome c oxidase subunits 1, 2 and 3
DNA: Deoxyribonucleic acid
kb: Kilo base pair
min: Minute
MRCA: Most recent common ancestor
mt: Mitochondrial
Mya: Million years ago
*nad1*, *nad2*, *nad3*, *nad4*, *nad4L*, *nad5* and *nad6*: Mitochondrial genes for NADH dehydrogenase subunits 1-6 and 4 L
PCR: Polymerase chain reaction
RNA: Ribonucleic acid
rRNA: Ribosomal RNA
*rrnS* and *rrnL*: Genes for small and large subunits of ribosomal RNA
sec: Second
T: Thymine
tRNA: Transfer RNA
*trnA* or *A*: tRNA gene for alanine
*trnC* or *C*: tRNA gene for cysteine
*trnD* or *D*: tRNA gene for aspartic acid
*trnE* or *E*: tRNA gene for glutamic acid
*trnF* or *F*: tRNA gene for phenylalanine
*trnG* or *G*: tRNA gene for glycine
*trnH* or *H*: tRNA gene for histidine
*trnI* or *I*: tRNA gene for isoleucine
*trnK* or *K*: tRNA gene for lysine
*trnL*_*1*_ or *L*_*1*_: tRNA gene for leucine (anticodon NAG)
*trnL*_*2*_ or *L*_*2*_: tRNA gene for leucine (anticodon YAA)
*trnM* or *M*: tRNA gene for methionine
*trnN* or *N*: tRNA gene for asparagine
*trnP* or *P*: tRNA gene for proline
*trnQ* or *Q*: tRNA gene for glutamine
*trnR* or *R*: tRNA gene for arginine
*trnS*_*1*_ or *S*_*1*_: tRNA gene for serine (anticodon NCU)
*trnS*_*2*_ or *S*_*2*_: tRNA gene for serine (anticodon NGA)
*trnT* or *T*: tRNA gene for threonine
*trnV* or *V*: tRNA gene for valine
*trnW* or *W*: tRNA gene for tryptophan
*trnY* or *Y*: tRNA gene for tyrosine
U: Uracil
