# Supplementary material for: Fragmented mitochondrial genomes of seal lice (family Echinophthiriidae) and gorilla louse (family Pthiridae): frequent minichromosomal splits and a host switch of lice between seals
Source: BMC Genomics. 2022 Apr 8;23:283. doi: 10.1186/s12864-022-08530-8 (PMC8994281; doi:10.1186/s12864-022-08530-8)
Supplement: Supplementary file 1 — Additional file 1: Supplementary Fig. 1. Secondary structure inferred with tRNA-Scan [33] from trnW1 and trnW2 gene sequences of the southern elephant seal louse, Lepidophthirus macrorhini. Supplementary Fig. 2. Conserved non-coding AT-rich motifs and GC-rich motifs among the mitochondrial minichromosomes of the southern elephant seal louse, Lepidophthirus macrorhini. Supplementary Fig. 3. Conserved non-coding AT-rich motifs and GC-rich motifs among the mitochondrial minichromosomes of the northern fur seal louse, Proechinophthirus fluctus. Supplementary Fig. 4. Conserved non-coding AT-rich motifs and GC-rich motifs among the mitochondrial minichromosomes of the Weddell seal louse, Antarctophthirus carlinii. Supplementary Fig. 5. Conserved non-coding AT-rich motifs and GC-rich motifs among the mitochondrial minichromosomes of the crabeater seal louse, Antarctophthirus lobodontis. Supplementary Fig. 6. Conserved non-coding GC-rich motifs among the mitochondrial minichromosomes of the Australian sea lion louse, Antarctophthirus microchir. Supplementary Fig. 7. Conserved non-coding AT-rich motifs and GC-rich motifs among the mitochondrial minichromosomes of the gorilla louse, Pthirus gorillae. Supplementary Fig. 8. Phylogenetic relationships among 21 species of sucking lice (Anoplura) inferred by maximum likelihood (ML) analysis of nucleotide sequences of five mitochondrial protein-coding genes. The elephant louse, Haematomyzus elephantis, was used as the outgroup. The ultrafast bootstrap support (%) / SH-aLRT support (%) were indicated near each node. Supplementary Fig. 9. Alignment of partial 18S rRNA gene, 28S rRNA gene and ef1-α gene sequences between Antarctophthirus carlinii and Antarctophthirus lobodontis. Supplementary Table 1. Mitochondrial minichromosomes of Lepidophthirus macrorhini - louse of southern elephant seal (Mirounga leonine). Supplementary Table 2. Sequence identities between trnW1 and trnW2 of the southern elephant seal louse, Lepidophthirus macro [file 12864_2022_8530_MOESM1_ESM.pdf]

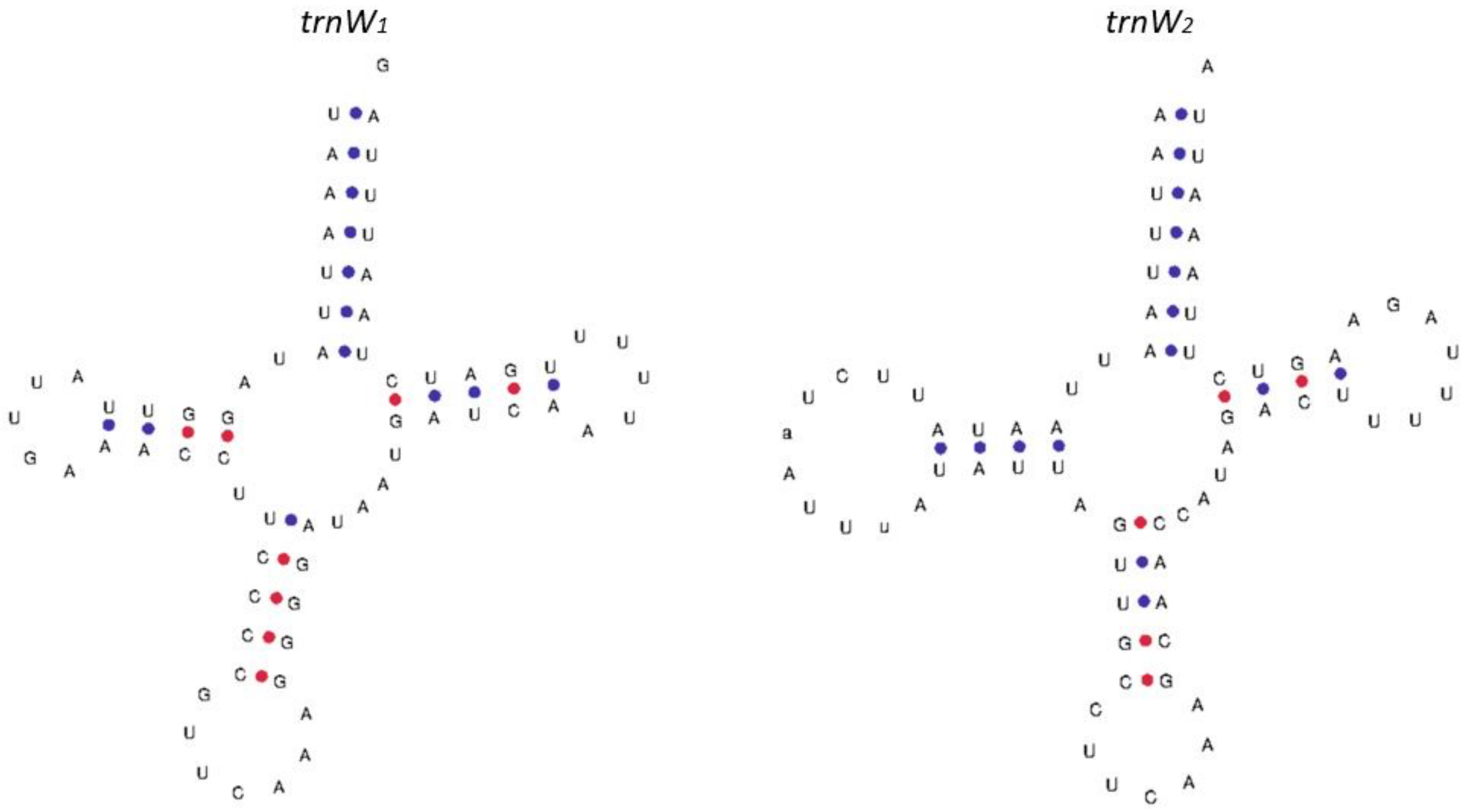


Supplementary Fig. 1. Secondary structure inferred with tRNA-Scan [33] from *trnW$_1$* and *trnW$_2$* gene sequences of the southern elephant seal louse, *Lepidophthirus macrorhini*.

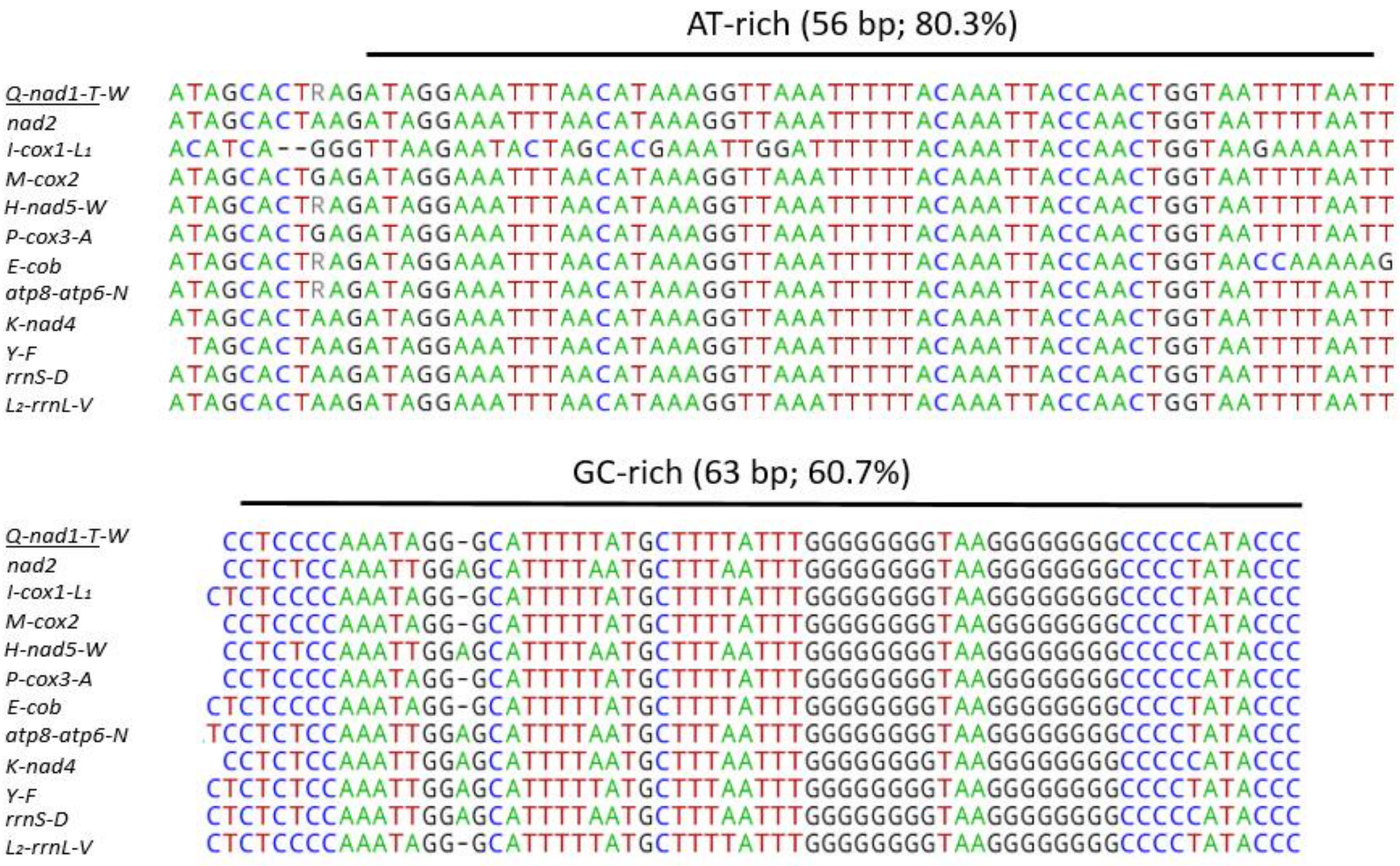


Supplementary Fig. 2. Conserved non-coding AT-rich motifs and GC-rich motifs among the mitochondrial minichromosomes of the southern elephant seal louse, *Lepidophthirus macrorhini.*

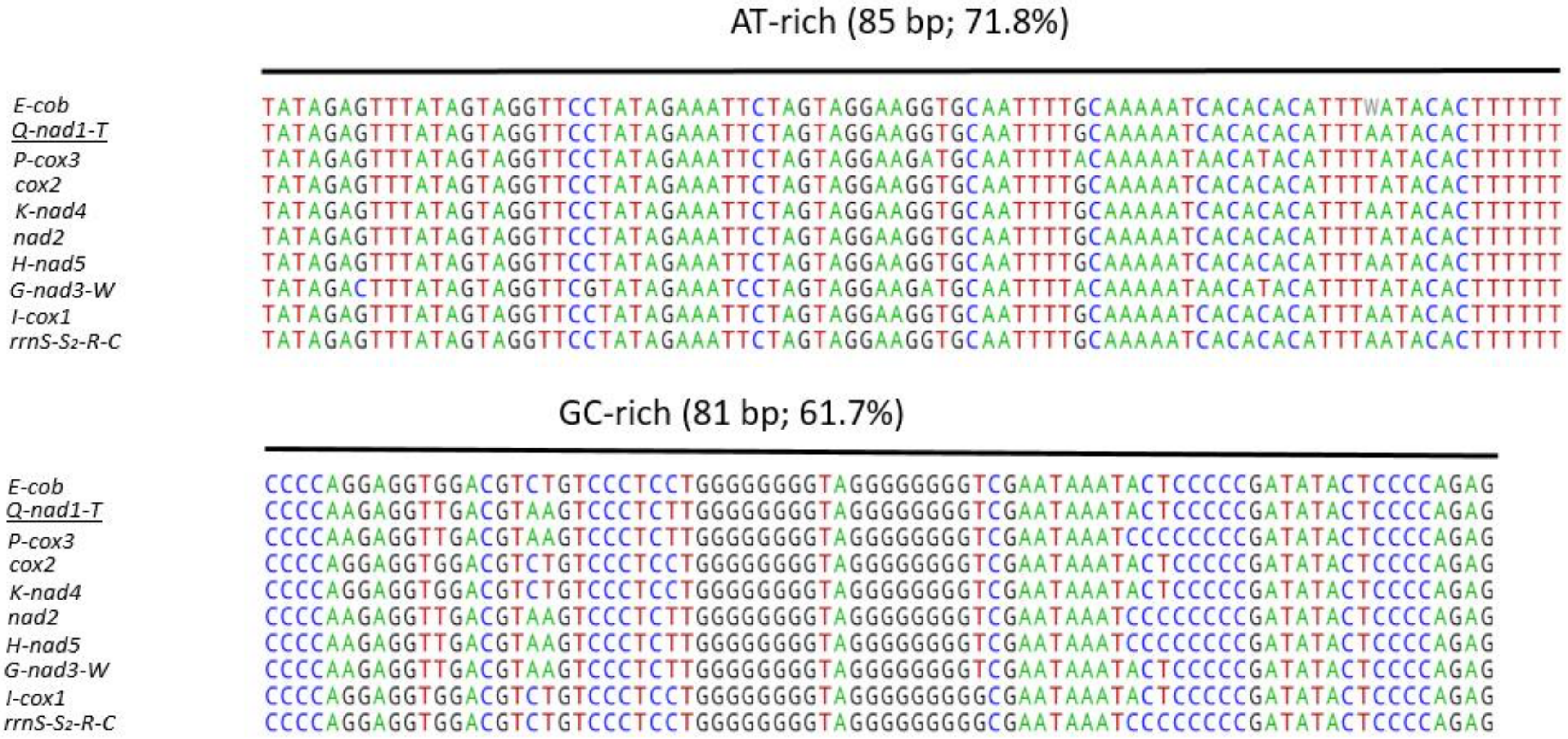


Supplementary Fig. 3. Conserved non-coding AT-rich motifs and GC-rich motifs among the mitochondrial minichromosomes of the northern fur seal louse, *Proechinophthirus fluctus*.

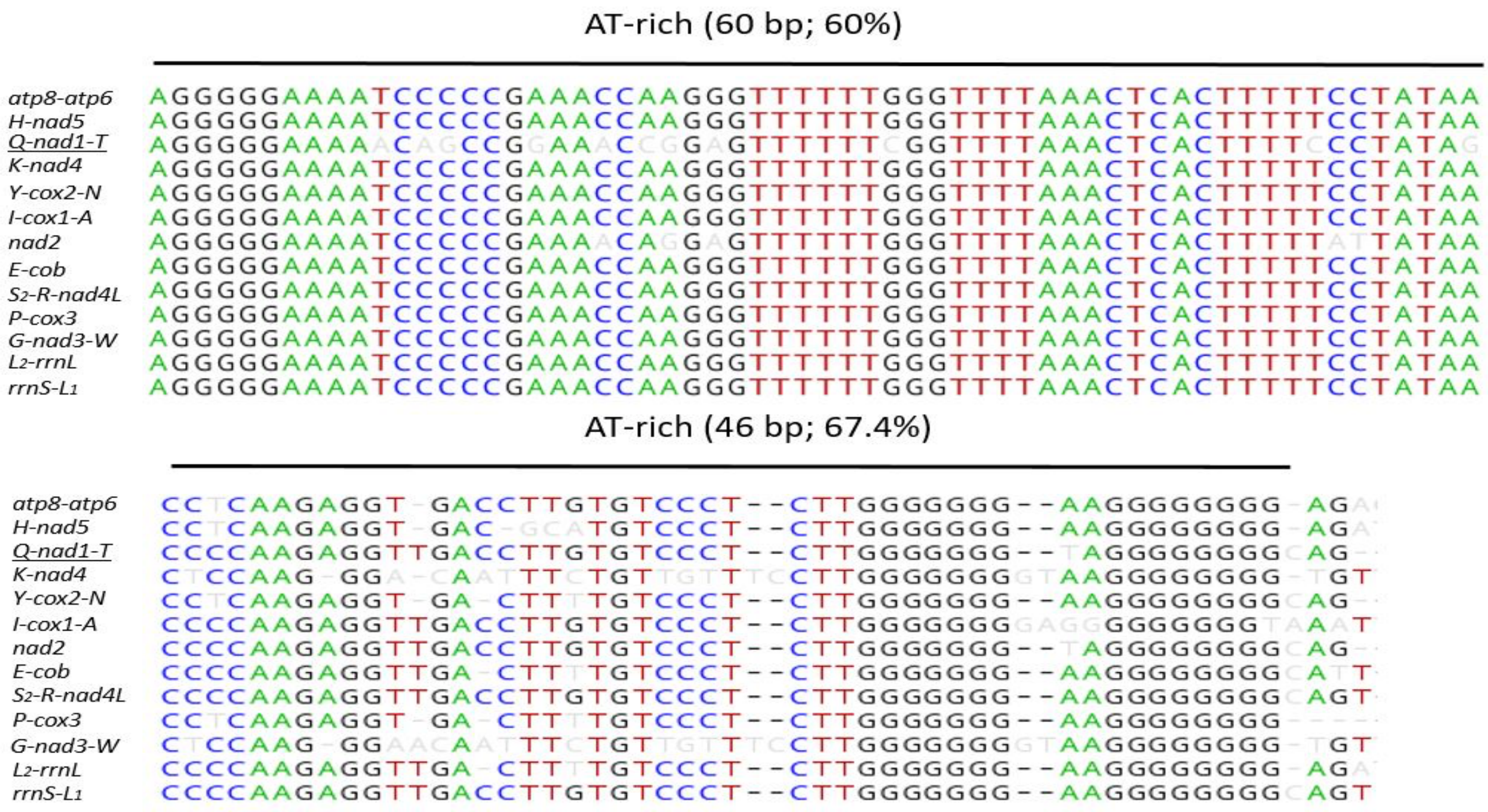

Supplementary Fig. 4. Conserved non-coding AT-rich motifs and GC-rich motifs among the mitochondrial minichromosomes of the Weddell seal louse, *Antarctophthirus carlinii.*

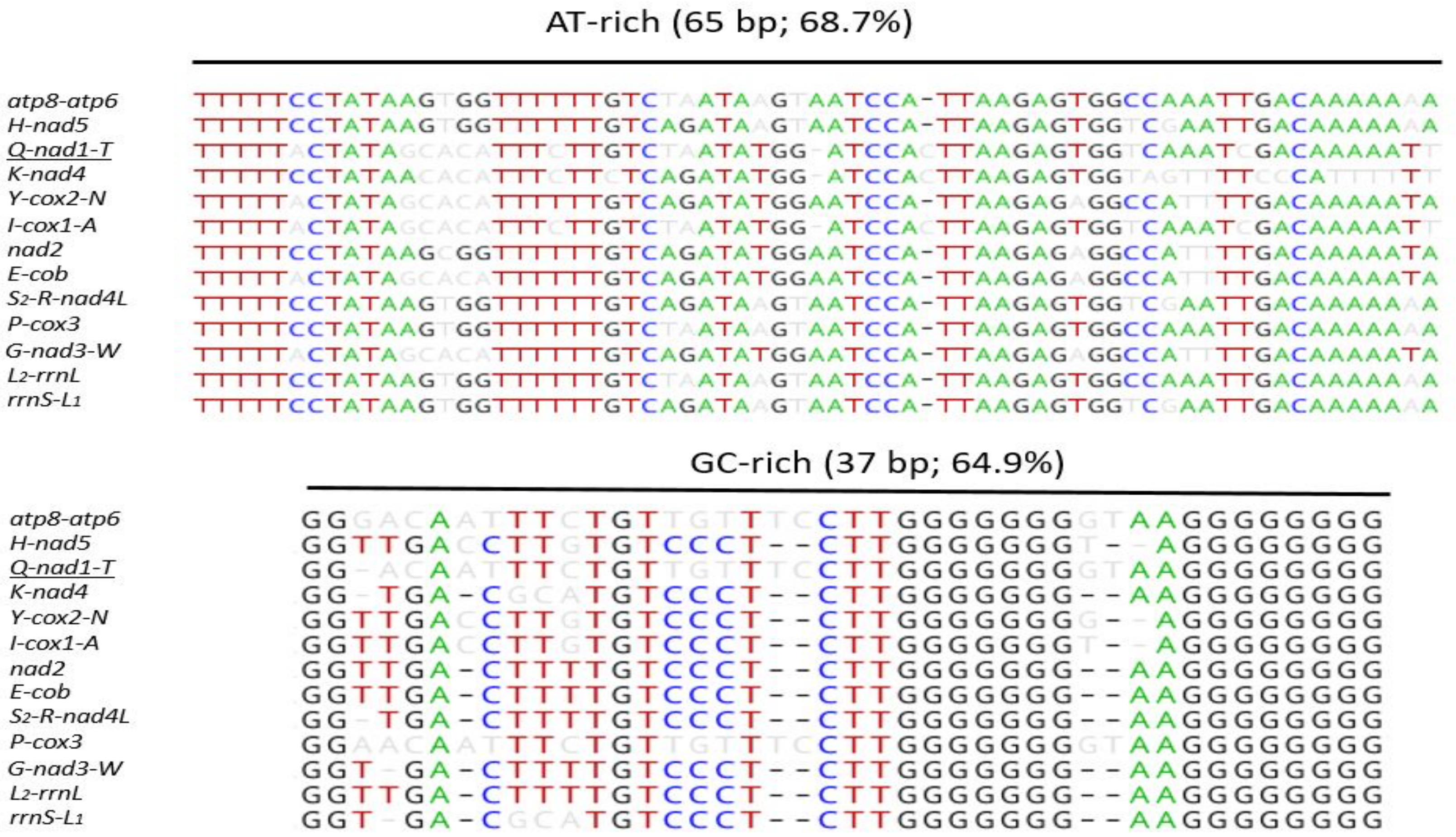


Supplementary Fig. 5. Conserved non-coding AT-rich motifs and GC-rich motifs among the mitochondrial minichromosomes of the crabeater seal louse, *Antarctophthirus lobodontis*.

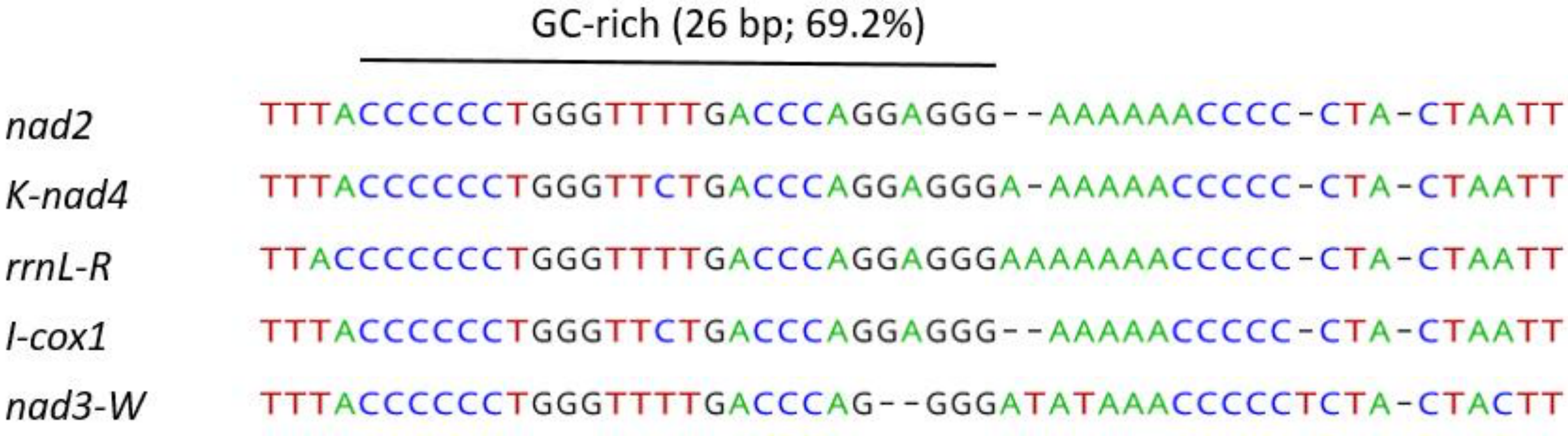


Supplementary Fig. 6. Conserved non-coding GC-rich motifs among the mitochondrial minichromosomes of the Australian sea lion louse, *Antarctophthirus microchir*.

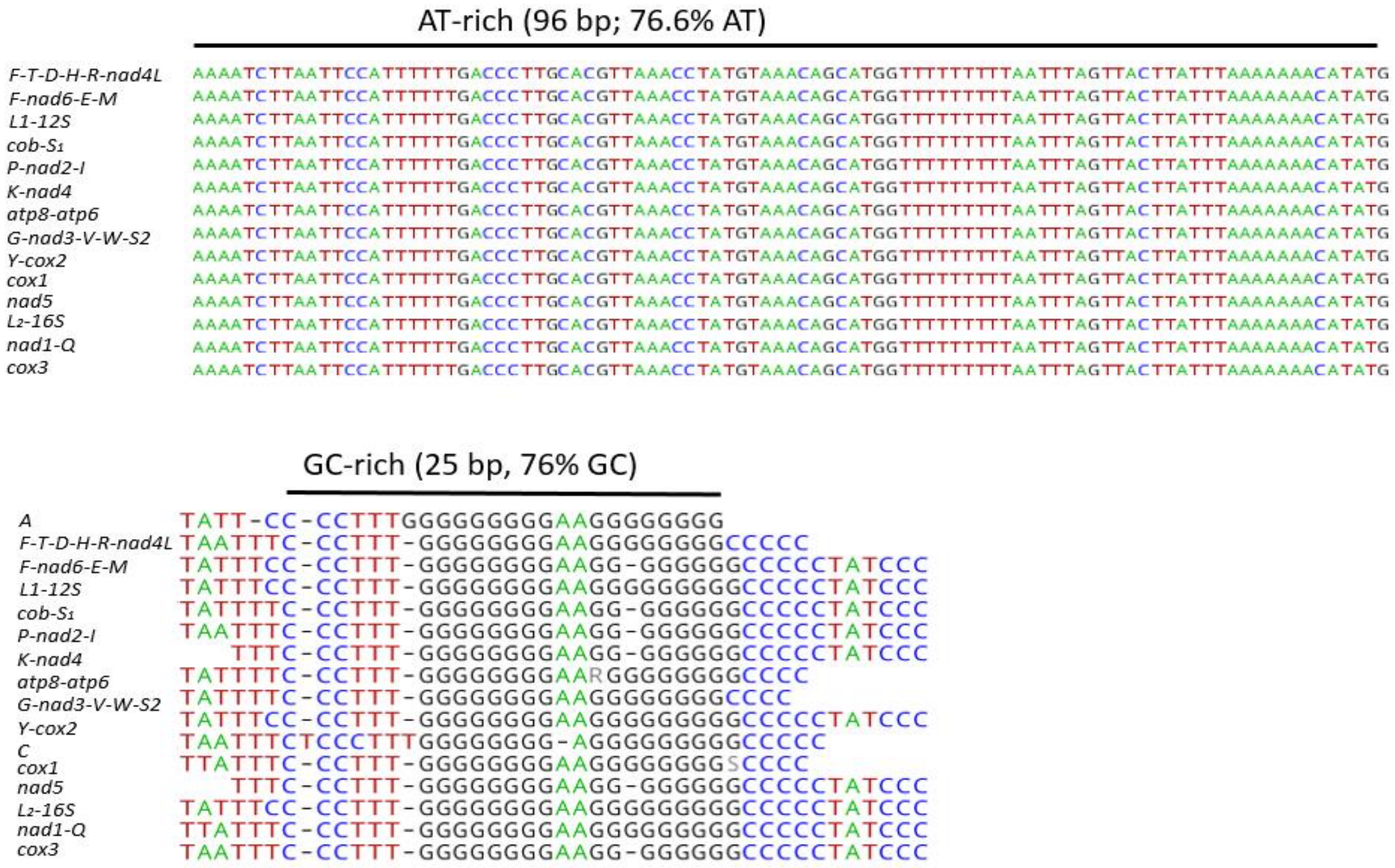


Supplementary Fig. 7. Conserved non-coding AT-rich motifs and GC-rich motifs among the mitochondrial minichromosomes of the gorilla louse, *Pthirus gorillae.*

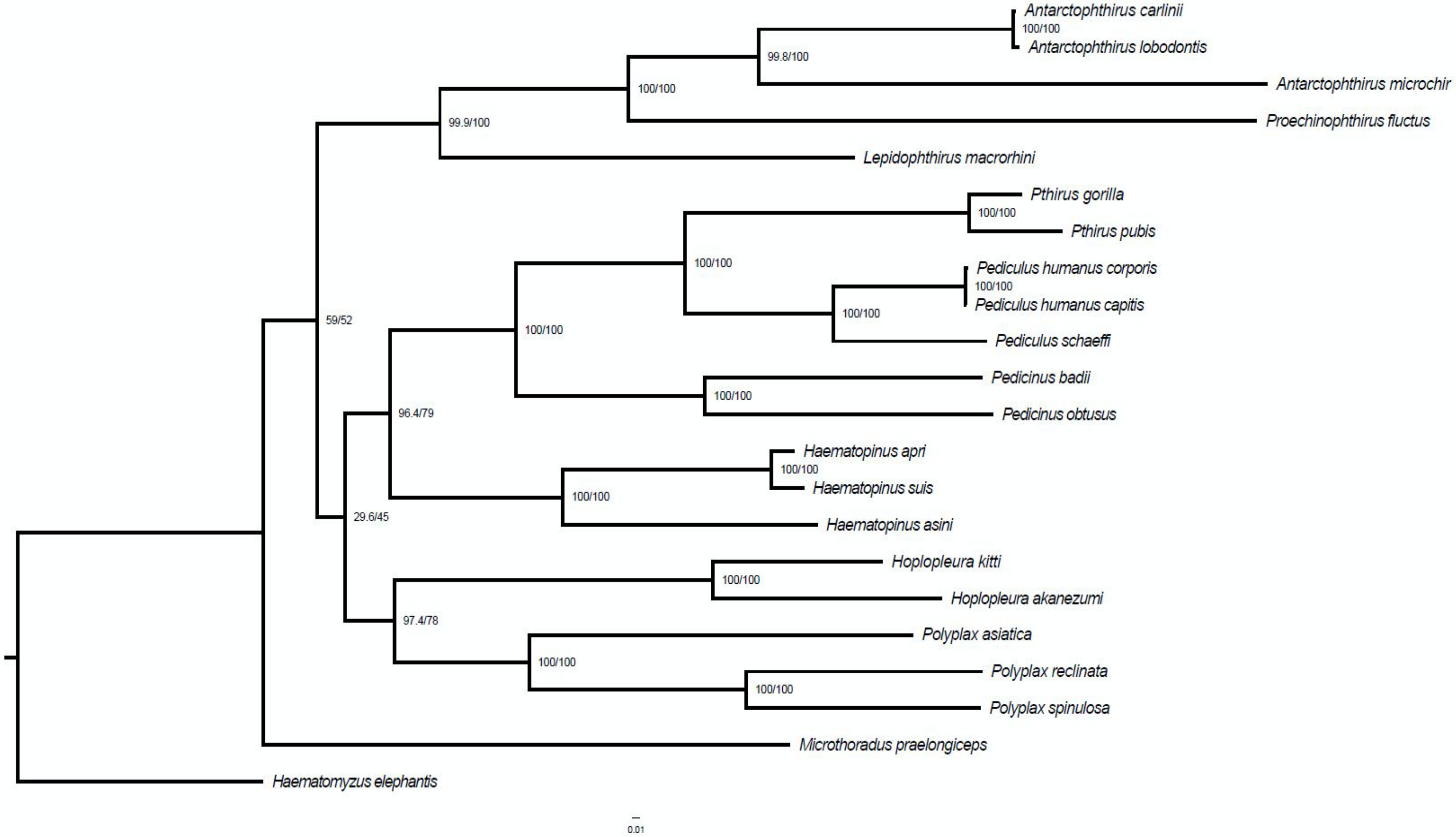


Supplementary Fig. 8. Phylogenetic relationships among 21 species of sucking lice (Anoplura) inferred by maximum likelihood (ML) analysis of nucleotide sequences of five mitochondrial protein-coding genes. The elephant louse, *Haematomyzus elephantis*, was used as the outgroup. The ultrafast bootstrap support (%) / SH-aLRT support (%) were indicated near each node.

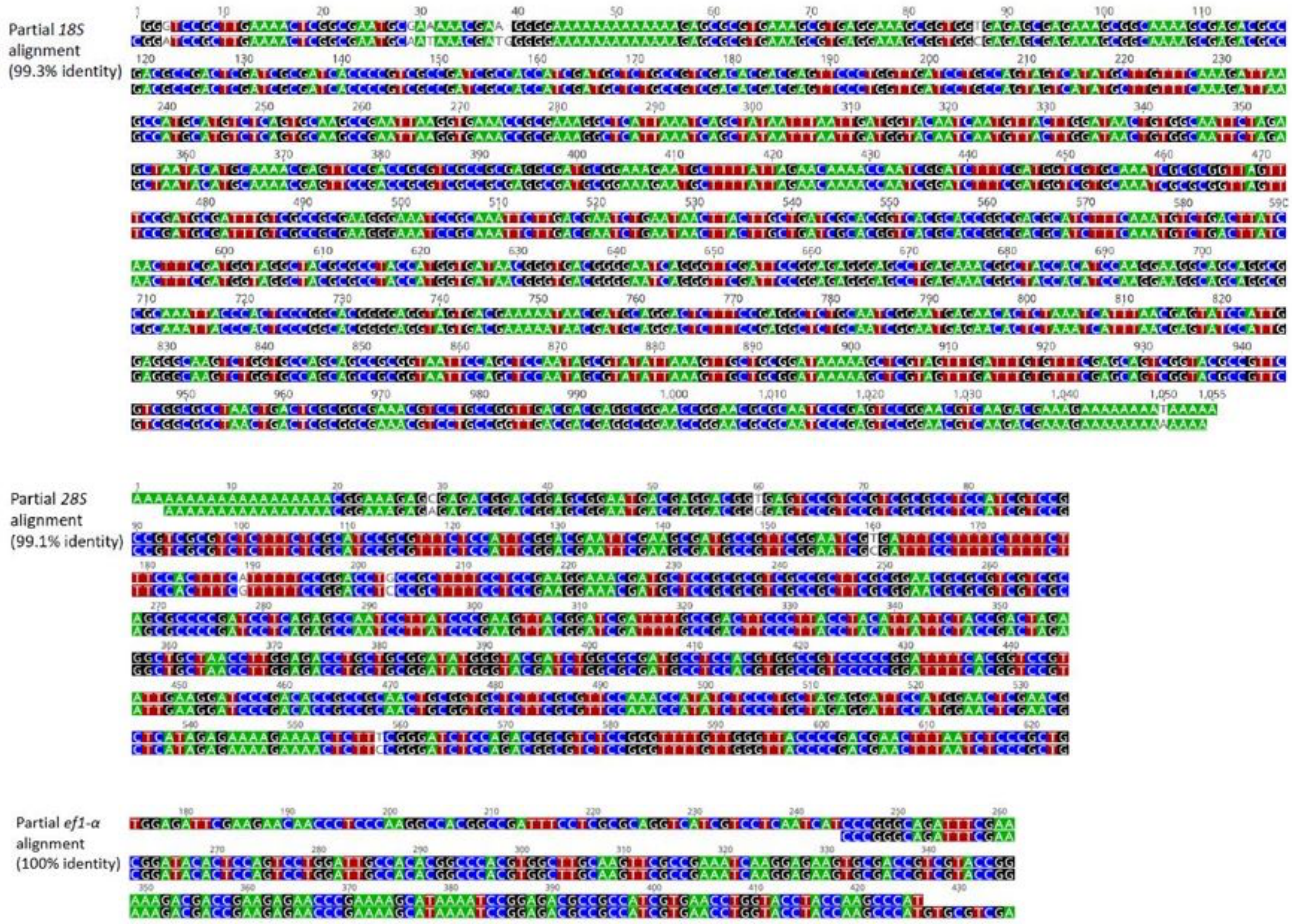


Supplementary Fig. 9. Alignment of partial *18S* rRNA gene, *28S* rRNA gene and *ef1-α* gene sequences between *Antarctophthirus carlinii* and *Antarctophthirus lobodontis*.

Supplementary Table 1. Mitochondrial minichromosomes of *Lepidophthirus macrorhini* - louse of southern elephant seal (*Mirounga leonine*)

| Minichromosome | Coding region size (bp) | Individual gene size (bp) | Assembled upstream non-coding region size (bp) | Assembled downstream non-coding region size (bp) | Number of Illumina sequence-reads | Mean coverage |
|---|---|---|---|---|---|---|
| *atp8-atp6-N* | 937 | 189-675-70 | 300 | 300 | 27366 | 1900 |
| *E-cob* | 1152 | 63-1089 | 300 | 300 | 23907 | 1720 |
| *I-cox1-L*$_1$ | 1668 | 66-1536-65 | 300 | 300 | 37607 | 2232 |
| *M-cox2* | 820 | 67-699 | 300 | 300 | 21714 | 2023 |
| *P-cox3-A* | 921 | 67-786-66 | 300 | 300 | 29089 | 2553 |
| *Q-nad1-T-W*$_1$ | 1152 | 67-64-921-68 | 300 | 300 | 60472 | 3586 |
| *nad2* | 1114 | 1047 | 300 | 300 | 71155 | 5801 |
| *K-nad4* | 1395 | 69-1326 | 300 | 293 | 53047 | 3752 |
| *H-nad5-W*$_2$ | 1891 | 66-1539-70 | 300 | 170 | 22603 | 1434 |
| *L*$_2$*-rrnL-V* | 1276 | 71-1139-66 | 300 | 273 | 75116 | 5679 |
| *rrnS-D* | 848 | 781-67 | 300 | 300 | 45830 | 4741 |

Supplementary Table 2. Sequence identities between *trnW*$_1$ and *trnW*$_2$ of the southern elephant seal louse, *Lepidophthirus macrorhini*, between *trnW* genes of *Lepidophthirus macrorhini* and other seal lice, and between *trnW* genes and other tRNA genes of *Lepidophthirus macrorhini*. Identities were generated with ClustalW in Geneious [29]: cost matrix IUB, gap open cost 15, gap extend cost 6.66.

| | ***trnW*$_1$** | ***trnW*$_2$** |
|---|---|---|
| ***trnW*$_2$** | 50% | |
| *trnW* (*Antarctophthirus carlinii*) | 68.7% | 55.1% |
| *trnW* (*Antarctophthirus lobodontis*) | 68.7% | 55.1% |
| *trnW* (*Antarctophthirus microchir*) | 69.1% | 42.6% |
| *trnW* (*Proechinophthirus fluctus*) | 55.4% | 48.5% |
| *trnA* | 44.8% | 44.8% |
| *trnD* | 50.7% | 50% |
| *trnE* | 48.4% | 50.9% |
| *trnH* | 50% | 48.5% |
| *trnI* | 47.8% | 47.8% |
| *trnK* | 50.7% | 53.6% |
| *trnL*$_1$ | 46.9% | 52.2% |
| *trnL*$_2$ | 49.2% | 43.7% |
| *trnM* | 51.5% | 42.6% |
| *trnN* | 50.7% | 53.5% |
| *trnP* | 48.5% | 38.8% |
| *trnQ* | 51.5% | 49.3% |
| *trnT* | 50% | 50% |
| *trnV* | 52.2% | 47.1% |

Supplementary Table 3. Mitochondrial minichromosomes of *Proechinophthirus fluctus* - louse of northern fur seal (*Callorhinus ursinus*)

| Minichromosome | Coding region size (bp) | Individual gene size (bp) | Assembled upstream non-coding region size (bp) | Assembled downstream non-coding region size (bp) | Number of Illumina sequence-reads | Mean coverage |
|---|---|---|---|---|---|---|
| *E-cob* | 1156 | 64-1092 | 200 | 200 | 23212 | 1495 |
| *I-cox1* | 1598 | 73-1503 | 200 | 200 | 26228 | 1314 |
| *cox2* | 765 | 765 | 200 | 264 | 24242 | 1997 |
| *P-cox3* | 849 | 67-783 | 200 | 200 | 22441 | 813 |
| *Q-nad1-T* | 1042 | 66-910-67 | 200 | 149 | 13414 | 915 |
| *nad2* | 1029 | 1029 | 200 | 200 | 34297 | 1370 |
| *G-nad3-W* | 481 | 67-345-65 | 200 | 200 | 4011 | 763 |
| *K-nad4* | 1379 | 70-1308 | 200 | 200 | 23312 | 1321 |
| *H-nad5* | 1739 | 60-1680 | 200 | 200 | 12873 | 631 |
| *rrnS*-$S_2$-*R-nad4L-C* | 1249 | 772-68-63-65 | 200 | 253 | 30866 | 1840 |

Supplementary Table 4. Mitochondrial minichromosomes of *Antarctophthirus carlinii* - louse of Weddell seal (*Leptonychotes weddelli*)

| Minichromosome | Coding region size (bp) | Individual gene size (bp) | Assembled upstream non-coding region size (bp) | Assembled downstream non-coding region size (bp) | Number of Illumina sequence-reads | Mean coverage |
|---|---|---|---|---|---|---|
| *atp8-atp6* | 829 | 151-672 | 300 | 300 | 21419 | 1840 |
| *E-cob* | 1157 | 65-1092 | 300 | 297 | 11440 | 1063 |
| *I-cox1-A* | 1670 | 66-1539-64 | 300 | 300 | 18669 | 1283 |
| *Y-cox2-N* | 805 | 65-674-65 | 300 | 300 | 8032 | 919 |
| *P-cox3* | 848 | 62-786 | 300 | 241 | 13456 | 1488 |
| *Q-nad1-T* | 1040 | 68-903-68 | 300 | 300 | 12074 | 1173 |
| *nad2* | 1056 | 1056 | 300 | 300 | 11297 | 1083 |
| *G-nad3-W* | 529 | 69-345-72 | 300 | 300 | 17009 | 1864 |
| *K-nad4* | 1373 | 67-1305 | 300 | 300 | 13739 | 1095 |
| *H-nad5* | 1756 | 70-1681 | 300 | 300 | 16301 | 1077 |
| *$S_2$-R-nad4L* | 420 | 70-69-279 | 300 | 300 | 8957 | 1098 |
| *$L_2$-rrnL* | 1421 | 64-1357 | 300 | 300 | 28669 | 1779 |
| *rrnS-$L_1$* | 908 | 839-69 | 300 | 300 | 19927 | 1858 |

Supplementary Table 5. Mitochondrial minichromosomes of *Antarctophthirus lobodontis* - louse of crabeater seal (*Lobodon carcinophagus*)

| Minichromosome | Coding region size (bp) | Individual gene size (bp) | Assembled upstream non-coding region size (bp) | Assembled downstream non-coding region size (bp) | Number of Illumina sequence-reads | Mean coverage |
|---|---|---|---|---|---|---|
| *atp8-atp6* | 829 | 151-672 | 300 | 300 | 27025 | 2567 |
| *E-cob* | 1157 | 65-1092 | 300 | 300 | 39824 | 3188 |
| *I-cox1-A* | 1670 | 66-1539-64 | 300 | 300 | 56989 | 3572 |
| *Y-cox2-N* | 807 | 65-674-69 | 300 | 300 | 32791 | 3233 |
| *P-cox3* | 848 | 62-786 | 300 | 241 | 30577 | 2977 |
| *Q-nad1-T* | 1040 | 68-903-68 | 300 | 277 | 29362 | 2527 |
| *nad2* | 1056 | 1056 | 300 | 300 | 31499 | 2645 |
| *G-nad3-W* | 529 | 69-345-72 | 300 | 300 | 22343 | 2641 |
| *K-nad4* | 1373 | 67-1305 | 300 | 300 | 44979 | 3228 |
| *H-nad5* | 1756 | 70-1686 | 300 | 300 | 44907 | 2719 |
| *$S_2$-R-nad4* | 420 | 70-69-279 | 300 | 300 | 17670 | 2322 |
| *$L_2$-rrnL* | 1429 | 64-1365 | 300 | 266 | 51799 | 3296 |
| *rrnS-$L_1$* | 866 | 797-69 | 300 | 300 | 24709 | 2293 |

Supplementary Table 6. Mitochondrial minichromosomes of *Antarctophthirus microchir* – louse of Australian sea lion (*Neophoca cinerea*)

| Minichromosome | Coding region size (bp) | Individual gene size(bp) | Assembled upstream non-coding region size (bp) | Assembled downstream non-coding region size (bp) | Number of Illumina sequence-reads | Mean coverage |
|---|---|---|---|---|---|---|
| *atp8-atp6-D* | 1083 | 180-645-67 | 250 | 250 | 79505 | 7055 |
| *cob* | 1215 | 1215 | 250 | 250 | 83187 | 7479 |
| *I-cox1* | 1607 | 65-1542 | 250 | 250 | 101204 | 6850 |
| *Y-cox2* | 792 | 69-723 | 250 | 250 | 47664 | 5050 |
| *P-cox3* | 908 | 68-840 | 250 | 250 | 56986 | 5541 |
| *nad1-T* | 991 | 82-909 | 250 | 250 | 76311 | 7167 |
| *nad2* | 996 | 996 | 250 | 250 | 63260 | 5917 |
| *nad3-W* | 384 | 312-68 | 250 | 250 | 30294 | 4601 |
| *K-nad4* | 1348 | 69-1278 | 250 | 250 | 71246 | 5459 |
| *nad5* | 1704 | 1704 | 250 | 250 | 85929 | 5561 |
| *rrnL-R* | 1443 | 1369-74 | 250 | 250 | 110797 | 9148 |
| *rrnS* | 731 | 731 | 250 | 250 | 74805 | 8256 |

Supplementary Table 7. Mitochondrial minichromosomes of *Pthirus gorilla* – louse of western gorilla (*Gorilla gorilla*)

| Minichromosome | Coding region size (bp) | Individual gene size (bp) | Assembled upstream non-coding region size (bp) | Assembled downstream non-coding region size (bp) | Number of Illumina sequence-reads | Mean coverage |
|---|---|---|---|---|---|---|
| *atp8-atp6* | 833 | 186-642 | 320 | 156 | 151286 | 17988 |
| *cob-$S_1$* | 1137 | 1071-64 | 320 | 316 | 165473 | 15005 |
| *cox1* | 1551 | 1551 | 320 | 159 | 169187 | 13484 |
| *Y-cox2* | 756 | 69-687 | 291 | 320 | 141626 | 16356 |
| *cox3* | 807 | 807 | 320 | 309 | 154255 | 17083 |
| *nad1-Q* | 970 | 897-59 | 320 | 320 | 163499 | 16013 |
| *P-nad2-I* | 1126 | 66-984-66 | 320 | 311 | 157520 | 14332 |
| *G-nad3-V-W-$S_2$* | 784 | 65-363-68-65-65 | 320 | 74 | 144350 | 18863 |
| *K-nad4* | 1338 | 68-1269 | 320 | 320 | 166965 | 13546 |
| *F-T-D-H-R-nad4L* | 859 | 65-66-70-68-65-276 | 320 | 320 | 137088 | 14455 |
| *nad5* | 1659 | 1659 | 320 | 320 | 183897 | 13054 |
| *F-nad6-E-M* | 749 | 65-489-64-67 | 320 | 199 | 135817 | 15392 |
| *$L_1$/$L_2$-rrnL* | 1288 | 68/68-1220 | 320 | 295 | 193756 | 16203 |
| *rrnS* | 819 | 67-752 | 257 | 320 | 135702 | 15075 |
| *A* | 65 | 65 | 320 | 44 | 8539 | 2478 |
| *C* | 64 | 64 | 320 | 34 | 427 | 129 |

Supplementary Table 8. Sequence identities between *Antarctophthirus carlinii* (louse of Weddell seal, *Leptonychotes weddelli*) and *Antarctophthirus lobodontis* (louse of crabeater seal, *Lobodon carcinophagus*)

| **Gene** | **Identity** | **Gene** | **Identity** |
|---|---|---|---|
| *atp8* | 100% | *trnG* | 100% |
| *atp6* | 98.1% | *trnH* | 100% |
| *cob* | 98.3% | *trnI* | 100% |
| *cox1* | 99.1% | *trnK* | 100% |
| *cox2* | 98.8% | *trn L*$_1$ | 100% |
| *cox3* | 98.5% | *trnL*$_2$ | 100% |
| *nad1* | 97.5% | *trnN* | 98.5% |
| *nad2* | 98.3% | *trnO* | 100% |
| *nad3* | 99.1% | *trnP* | 100% |
| *nad4* | 98.7% | *trnR* | 98.6% |
| *nad4L* | 98.9% | *trn S*$_2$ | 100% |
| *nad5* | 98.1% | *trnT* | 97.1% |
| *rrnL* | 98.9% | *trnW* | 100% |
| *rrnS* | 94.3% | *trnY* | 100% |
| *trnA* | 100% | Average identity | 99.03% |
| *trnE* | 100% | | |

Supplementary Table 9. Sequence identities between Weddell seal (*Leptonychotes weddelli*) and crabeater seal (*Lobodon carcinophagus*)

| **Gene** | **Identity** | **Gene** | **Identity** |
|---|---|---|---|
| *atp8* | 94.1% | *atp6* | 91.2% |
| *cob* | 92.5% | *cox1* | 93% |
| *cox2* | 93.7% | *cox3* | 92.2% |
| *nad1* | 94% | *nad2* | 92.4% |
| *nad3* | 90.8% | *nad4* | 91.5% |
| *nad4L* | 93.9% | *nad5* | 91.6% |
| *nad6* | 93.4% | *rrnS* | 96.1% |
| *rrnL* | 96.4% | *trnA* | 98.6% |
| *trnC* | 95.5% | *trnD* | 94% |
| *trnE* | 97.1% | *trnF* | 97.2% |
| *trnG* | 98.5% | *trnH* | 100% |
| *trnI* | 95.7% | *trnK* | 92.8% |
| *trnL1* | 97.2% | *trnL2* | 97.3% |
| *trnM* | 100% | *trnN* | 93.2% |
| *trnP* | 95.5% | *trnQ* | 91.8% |
| *trnR* | 95.7% | *trnS1* | 98.5% |
| *trnS2* | 96.6% | *trnT* | 92.9% |
| *trnV* | 94.1% | *trnW* | 97% |
| *trnY* | 98.5% | Average identity | 94.99% |

Supplementary Table 10. Species of parasitic lice included in the phylogenetic analyses in this study

| Species | Host | GenBank accession number | References |
|---|---|---|---|
| *Antarctophthirus microchir* | Sea lion | MW803082-93 | Present study |
| *Antarctophthirus carlinii* | Weddell seal | MW803073-81 | Present study |
| *Antarctophthirus lobodontis* | Crabeater seal | MW803064-72 | Present study |
| *Lepidophthirus macrorhini* | Elephant seals | MW803094-104 | Present study |
| *Proechinophthirus fluctus* | Fur seal | MW803105-114 | Present study |
| *Pthirus gorilla* | Gorilla | MW803115-131 | Present study |
| *Pedicinus obtusus* | Macaque | MT792495-506 | Fu & Dong et al., 2020 |
| *Pedicinus badii* | Colobus | MT721726-39 | Fu & Dong et al., 2020 |
| *Pediculus humanus corporis* | Human | FJ499473-90 | Shao et al., 2009 |
| *Pediculus humanus capitis* | Human | JX080388-407 | Shao et al., 2012 |
| *Pediculus schaeffi* | Chimpanzee | KC241882-97, KR706168-69 | Herd et al., 2015 |
| *Pthirus pubis* | Human | JQ976018, EU219987-95, HM241895-8, MT721740 | Shao et al., 2012; |
| *Polyplax asiatica* | Greater bandicoot rat | KF647751-61 | Dong et al., 2014b |
| *Polyplax reclinata* | Asian grey shrew | MW291451-61 | Dong et al., 2021 |
| *Polyplax spinulosa* | Asian house rat | KF647762-72 | Dong et al., 2014b |
| *Hoplopleura kitti* | Bower's white toothed rat | KJ648933-43 | Dong et al., 2014a |
| *Hoplopleura akanezumi* | Chevrier's field mouse | KJ648923-32 | Dong et al., 2014a |
| *Haematopinus apri* | Wild pig | KC814611-19 | Jiang et al., 2013 |
| *Haematopinus suis* | Domestic pig | KC814602-10 | Jiang et al., 2013 |
| *Haematopinus asini* | Horse | KF939318, KF939322, KF939324, KF939326, KJ434034-38 | Song et al., 2014 |
| *Microthoradus praelongiceps* | Guanacos | KX090378-KX090389 | Shao et al., 2017 |
| *Haematomyzus elephantis* | Elephant | KF933032- KF933041 | Shao et al., 2015 |
